# Global assessment of hepatic safety in novel immunotherapies: a systematic review and meta-analysis

**DOI:** 10.3389/fimmu.2025.1677998

**Published:** 2026-01-12

**Authors:** Minyan Ye, Yinuo Dong, Xiaoyun Li, Yang Zhi, Yuping Lu, Jieting Tang, Wei Zhong, Xiaohong Lei, Yimin Mao, Sha Huang, Yanyan Song

**Affiliations:** 1 Department of Medical Oncology, Clinical Oncology School of Fujian Medical University, Fujian Cancer Hospital, Fuzhou, Fujian, China; 2 Division of Gastroenterology and Hepatology, Shanghai Institute of Digestive Diseases, National Health Commission (NHC) Key Laboratory of Digestive Diseases, Shanghai Research Center of Fatty Liver Disease, Renji Hospital, School of Medicine, Shanghai Jiao Tong University, Shanghai, China; 3 Department of Hepatology, Hepatology Research Institute, The First Affiliated Hospital, Fujian Medical University, Fujian Clinical Research Center for Liver and Intestinal Diseases, Fuzhou, Fujian, China; 4 Department of Biostatistics, Clinical Research Institute, Shanghai Jiaotong University School of Medicine, Shanghai, China

**Keywords:** advanced effect, immunotherapy, LAG-3, meta - analysis, safety, TIGIT

## Abstract

**Background:**

This study explored whether integrating innovative immunotherapies targeting costimulatory or co-inhibitory pathways beyond standard PD-1, PD-L1, and CTLA-4 treatments affects hepatic adverse events. We further analyzed liver-related side effects in patients with cancer receiving these novel therapies alone or in combination with others.

**Methods:**

Clinical studies on immunotherapies targeting molecules such as LAG-3, TIGIT, TIM-3, VISTA, CD47, ICOS, CD40, and B7-H3 were retrieved from PubMed, Embase, Cochrane Library, and Web of Science. Data from eligible studies that reported liver-related adverse events until May 2024 were included.

**Results:**

This analysis included 63 studies involving 7,327 patients. Among these, randomized controlled trials demonstrated that adding LAG-3 or TIGIT inhibitors to established therapies did not increase the risk of elevated hepatic enzyme levels or hepatitis. CD27-CD70-targeted monotherapy showed a strong association with elevated transaminase levels. Dual therapies combining 4-1BB agonists with PD-1/PD-L1 inhibitors resulted in >15% all-grade transaminase elevation, whereas CD40 agonists paired with immunotherapy resulted in >4% high-grade elevations. Immunotherapy-chemotherapy combinations showed high transaminase elevation rates. Overall, the incidence of elevated liver enzyme levels was similar between the single-agent and dual immunotherapy groups. The addition of chemotherapy or targeted therapy to single-agent immunotherapy increases the incidence of adverse events associated with elevated liver enzyme levels. The incidence of liver enzyme adverse events continued to increase with the addition of immunotherapy to the combination regimens. Cholestatic enzyme elevations were prominent in CD27-CD70 monotherapy and CD40 agonist combinations.

**Conclusions:**

This meta-analysis suggests adding LAG-3 or TIGIT inhibitors to existing therapies may not significantly increase hepatic toxicity. It reviewed adverse events from novel immunotherapies alone or combined with PD-1/PD-L1/CTLA-4 inhibitors, targeted agents, or chemotherapy. These findings have important clinical implications.

## Introduction

In recent years, the FDA has approved multiple PD-1/PD-L1 and CTLA-4 inhibitors for the treatment of various solid tumors, including malignant melanomas. Immune checkpoint inhibitors (ICIs) targeting checkpoint receptors have established a pivotal role in cancer immunotherapy, and are increasingly being applied to tumors previously deemed refractory. In the treatment of hepatocellular carcinoma, bispecific antibodies targeting PD-1/TGF-β and PD-L1/VEGF have demonstrated clinically meaningful efficacy in clinical trials (1), three pivotal clinical trials—KEYNOTE-522, IMpassion031, and GeparNUEVO—have established immune checkpoint inhibitors (ICIs) as breakthrough therapies in the neoadjuvant treatment of early-stage triple-negative breast cancer (TNBC) (2), while these therapies have markedly enhanced survival rates, they also carry risks of immune dysregulation, manifesting as immune-related adverse events (irAEs) across multiple organ systems. These events frequently involve the skin, colon, endocrine glands, lungs, and, notably, the liver, which is a major target of immune-related toxicity (3). Hepatic adverse events are influenced by the type and dosage of ICIs. Although liver toxicity often presents as clinically mild, it may progress to severe outcomes including liver failure (4) or death (5, 6). High-grade hepatic irAEs were correlated with poor prognoses, underscoring the critical need for vigilant monitoring and management of liver function during immunotherapy.

The limitations of immunotherapy, such as resistance and treatment-related adverse events, often result in tumor progression. This is attributed to the loss of immunogenic neoantigens, accumulation of immunosuppressive cells, and upregulation of alternative immune checkpoint receptors (7). To address these challenges, combination therapies with enhanced efficacy have emerged as clinical strategies, although they may increase the risk of adverse events. Preventing resistance and managing these events remain central goals in cancer immunotherapy, and targeting additional immune checkpoints in tumors is considered a promising approach. Costimulatory and co-inhibitory pathways are crucial in regulating T cell activation and have gained attention for their roles in transforming treatment landscapes for refractory malignancies. Immune checkpoint inhibitors, such as PD-1, PD-L1, and CTLA-4, have been extensively studied, while newer agents targeting co-inhibitory molecules, including LAG-3, TIGIT, and TIM-3, are reshaping the clinical landscape. Key trials in this area include NCT02720068, NCT03598608, and NCT05064059. The approval of Relatlimab, a LAG-3 inhibitor, combined with nivolumab for metastatic melanoma in 2022 (8), highlights the clinical potential of such therapies. Furthermore, activation of costimulatory molecules (e.g., 4-1BB, OX40, GITR, and ICOS) enhances antitumor immune responses, as evidenced by NCT04740424 and NCT02904226.

Despite these advancements, the hepatic toxicity profile of these agents remains underexplored. While some studies have reported hepatic adverse events associated with LAG-3 inhibitors, TIGIT inhibitors, and their combination with PD-1 inhibitors in solid tumors such as colorectal cancer and melanoma (9–11), the majority of clinical trials involving novel immunotherapies targeting TIGIT, TIM-3, ICOS, and CD47 are still ongoing, and comprehensive reporting of related hepatotoxicity remains limited (12). There is a notable lack of reports specifically focused on the hepatotoxicity of these emerging immunotherapeutic agents.

A comprehensive evaluation of hepatic adverse events is essential to better understand the risks associated with both monotherapy and combination regimens, especially in comparison with existing treatments. Therefore, this review aims to summarize the incidence of liver-related adverse events associated with novel immunotherapies across various cancer types and treatment modalities, and to explore whether they confer an increased risk of hepatotoxicity compared to conventional therapies.

## Methods

This study was conducted in accordance with the Preferred Reporting Items for Systematic Reviews and Meta-Analyses (PRISMA) statement (13). A literature search was performed using PubMed、Embase、Cochrane Library 和 Web of Science databases. The study selection and data extraction were independently performed by three authors (XL, YS, JT). Any discrepancies were reviewed by a third investigator on the team, YM, and resolved through consensus.

### Search strategy

A systematic search of the PubMed, Embase, Cochrane Library, and Web of Science databases was performed to identify prospective clinical studies published in English concerning novel immune checkpoint inhibitors in advanced tumors up to May 1, 2024. The search encompassed presentations and abstracts from meetings of the American Society of Clinical Oncology (ASCO) and the European Society for Medical Oncology (ESMO) meetings. The key search terms used included “Carcinoma,” “LAG-3,” “TIGIT,” “TIM-3,” “PVRIG,” “CD112R,” “VISTA,” “BTNL2,” “BTN3A1,” “BTN2A1,” “BTLA,” “NKG2A,” “CD47,” “41BB,” “CD137,” “OX40,” “TNFRSF4,” “CD134,” “ICOS,” “CD278,” “CD40,” “CD28,” “CD27,” “GITR,” “4-1BB,” “B7-H3,” “B7-H4,” and “clinical trials.” In total, 9,139 articles were retrieved: 2,106 from PubMed, 791 from the Cochrane Library, 4,443 from Web of Science, and 1,799 from Embase, as summarized in **Supplementary Table 2**.

### Inclusion and exclusion criteria

**Figure 1:**
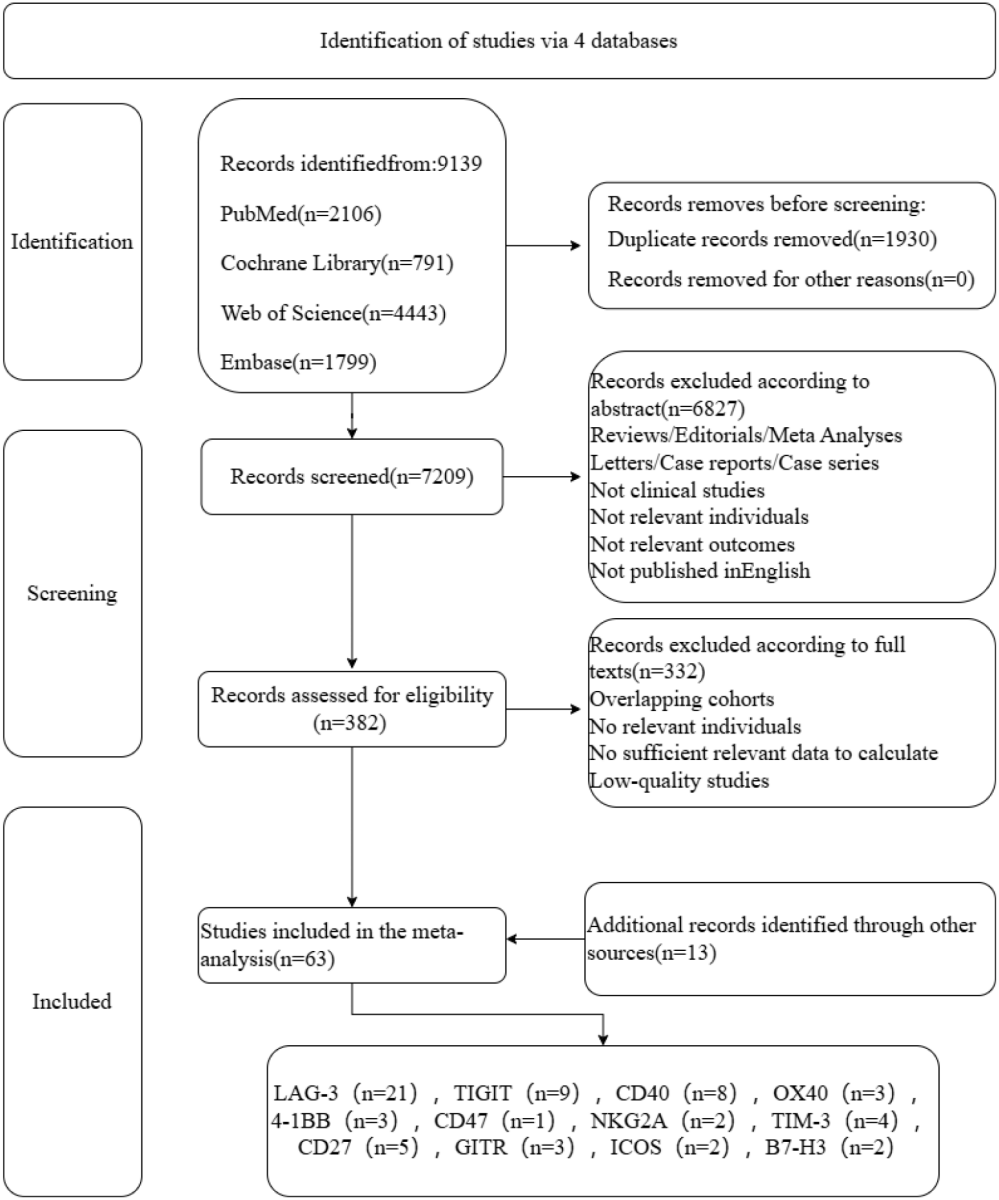
Diagram of the study selection process.

1. Clinical trials related to cancer treatment; 2. Novel immunotherapeutics targeting costimulatory or coinhibitory molecules, excluding PD-1, PD-L1, and CTLA-4; 3. Prospective single-arm studies or randomized controlled trials; 4. Articles published in English. Exclusion criteria: 1. Nonclinical studies, reviews, letters, pathology studies, systematic reviews, meta-analyses, case reports, case series, etc.; 2. articles published in languages other than English; 3. studies lacking relevant data on hepatic adverse events, 4. Studies involving unrelated populations, encompassing non-oncological patients, individuals not treated per protocol, and those lost to follow-up, were excluded; 5. Inaccessible original articles. Following the removal of 1,930 duplicate entries, 13 additional studies were identified from other sources (**Figure 1**).

### Literature screening and data extraction

The extracted key data included the trial name, NCT number, publication year, primary author, trial phase, randomization, blinding, treatment groups, therapy lines, and CTCAE version. The participant data included the total number and those in the safety analysis. Drug information included agent name, type, dosage, combination, and concomitant medications. Adverse events (AEs) included type, reporting standards, all-grade, and grade 3+ data. **Supplementary Table 3** summarizes the main study characteristics based on target location.

Outcomes: The primary goal of this study was to assess the impact of combining novel immune therapies with traditional anticancer treatments on liver-related adverse events. Additionally, this study aimed to summarize the incidence of adverse liver events associated with each new targeted agent. The occurrence rate of adverse events was calculated by dividing the number of participants affected by the total number of participants included in the safety analysis.

### Risk of bias and quality assessments

The methodological quality and risk of bias of the randomized controlled trials (RCTs) were assessed via the Jadad scale (14), which evaluates four key criteria: generation of random sequences, allocation concealment, blinding, and attrition/exclusion (**Supplementary Table 4**). For non-randomized cohort studies, the Newcastle–Ottawa Scale was employed (15), with scores ranging from 0 to 9 (**Supplementary Table 5**). Two authors (Y.L. and M.Y.) independently conducted this evaluation and any discrepancies in scoring were resolved by a third researcher (Y.M.).

### Statistical analysis

In accordance with the E3 Guideline of the International Council for Harmonization of Technical Requirements for Pharmaceuticals for Human Use (ICH), the incidence of hepatic adverse events (AEs) was calculated based on the total number of treated patients and the occurrence of specific biochemical abnormalities, including transaminase and cholestatic enzyme elevation. Risk differences with 95% confidence intervals (CIs) were calculated, and study heterogeneity was assessed using τ², I², H², and Cochran’s Q test (α = 0.10). Low heterogeneity was defined as I² < 25%, moderate heterogeneity as 25%-50%, and high heterogeneity as >50%. Random-effects models were used to generate forest plots for meta-analysis. Subgroup analyses stratified by target mechanism (LAG-3 *vs*. TIGIT inhibitors) and treatment modality were performed using random-effects models with relative risk (RR) and corresponding 95% confidence intervals (CIs) as summary statistics to quantify the treatment effects on both all-grade (1-5) and high-grade (3-4) AEs. An RR <1.0, indicating a safety benefit favoring the control arm, with two-sided p-values <0.05 considered statistically significant. The inverse variance method estimated single-arm proportions, whereas funnel and contour-enhanced funnel plots assessed small-study effects associated with publication bias. The Begg’s test further examined publication bias (**Supplementary Figure 1**, **Supplementary Table 1**). Statistical analyses were performed using the R software (version 4.1.1; R Foundation for Statistical Computing). All data processing, statistical modeling, and visualization were conducted using R’s core functionality and specialized packages, including list-relevant packages such as meta, metaprop, metabin, and ggplot2.

### Patient and public involvement

This study was conducted without patient involvement at any stage in the research process. The patients were not engaged in formulating the research question, determining the outcome measures, or contributing to the study design and implementation protocols. Furthermore, patient input was not required for data interpretation or for manuscript preparation. The dissemination plan does not include sharing research findings with study participants or a broader patient population. Notably, this systematic review did not assess the extent of patient involvement in the included studies.

## Results

### Literature screening results and research characteristics

A comprehensive review identified 9,139 records refined to 63 studies with 7,327 participants. The targets were LAG-3, TIGIT, TIM-3, CD47, NKG2A, 4-1BB, OX40, ICOS, CD27, GITR, CD40, and B7-H4. Therapeutic approaches included monotherapy (22 groups), combination therapy with PD-1/PD-L1/CTLA-4 inhibitors (53 groups), novel immunotherapy with targeted therapy or chemotherapy (14 groups), and dual immunotherapy (8 groups). Most studies focused on solid tumors, including melanoma, renal cell carcinoma, gastric, gallbladder, colon cancer, and mesothelioma, with 12 RCTs and 3 clinical investigations specific to hepatocellular carcinoma. Hepatotoxicity was assessed based on ALT, AST, ALP, and GGT levels, which were categorized as high-grade or all-grade elevations.

### Clinical trials of traditional antitumor therapies combined with novel immune therapies

To evaluate whether novel immunotherapy increases hepatic adverse events, we analyzed six RCTs, including three conference abstracts. Of these, three studies used a double-blind design (details in **Supplementary Table 3**). Statistical comparisons showed that adding LAG-3 inhibitors to PD-1 inhibitors or chemotherapy, or combining TIGIT inhibitors with PD-L1 inhibitors, chemotherapy, or VEGF inhibitors, did not significantly impact all-grade or high-grade hepatotoxicity (*p* > 0.05). These results suggest that LAG-3 and TIGIT inhibitors do not notably exacerbate hepatotoxicity.

**Figure 2:**
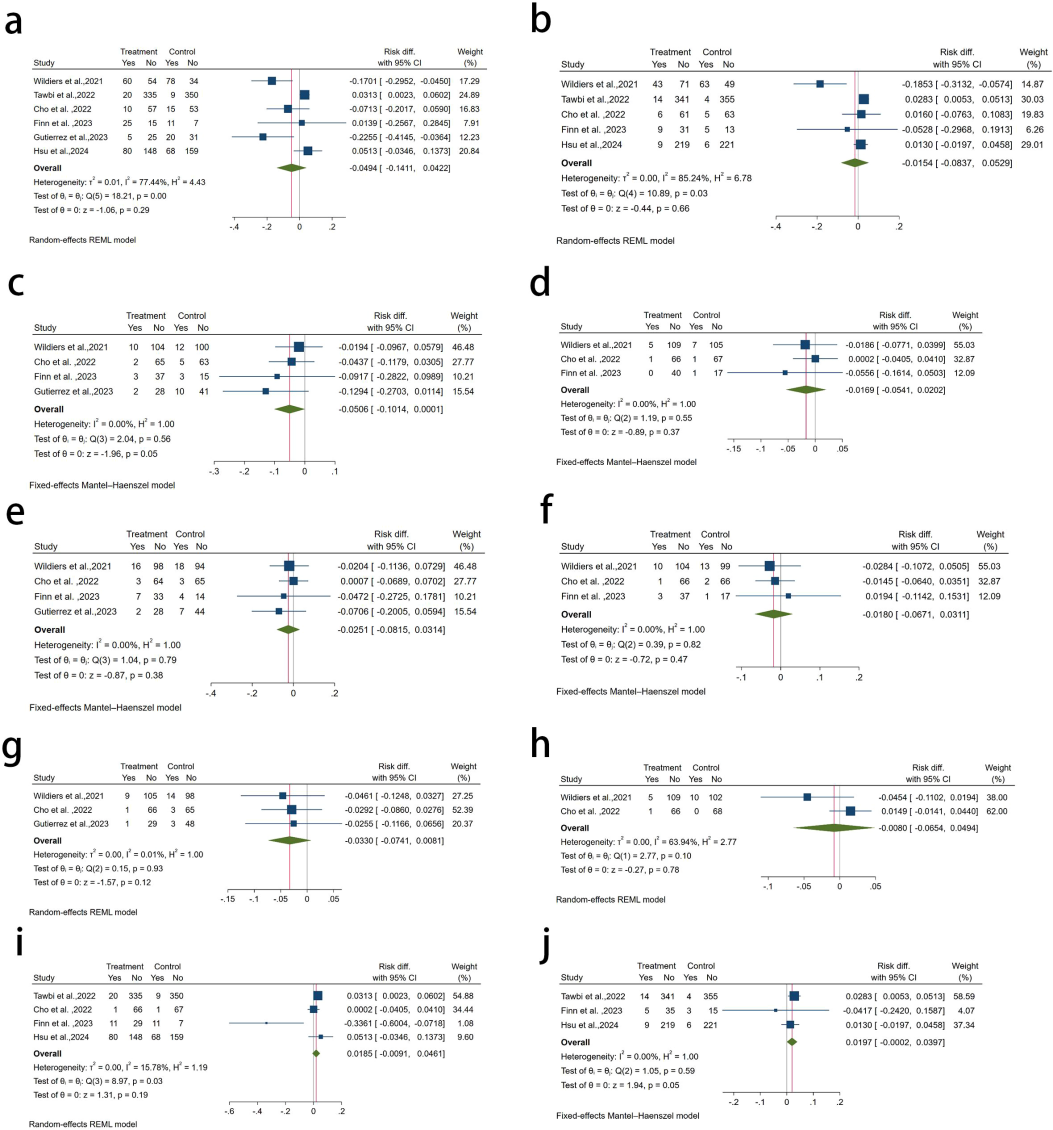
**(a)** all-grade hepatic adverse events; **(b)** grade ≥3 hepatic adverse events; **(c)** all-grade ALT increase; **(d)** grade ≥3 ALT increase; **(e)** all-grade AST increase; **(f)** grade ≥3 AST increase; **(g)** all-grade ALP increase; **(h)** grade ≥3 ALP increase; **(i)** all-grade hepatitis; **(j)** grade ≥3 hepatitis.

We also compared the treatment and control groups for the incidence of all-grade and high-grade elevations in ALT, AST, and ALP levels as well as the occurrence of hepatitis. The results suggested that neither LAG-3 nor TIGIT inhibitors significantly increased hepatic toxicity compared with traditional antineoplastic therapies (all-grade hepatic adverse events: -4.94% (95% CI, -14.11%, 4.22%), *p* > 0.05; b grade≥3 hepatic adverse events: -1.54% (95% CI, -8.37%, 5.29%), *p* > 0.05; c all-grade ALT increase: -5.06% (95% CI, -10.14%, 0.10%), *p* > 0.05; d grade ≥3 ALT increase:-1.69% (95% CI, -5.41%, 2.02%), *p>*0.05; e all-grade AST increase: -2.51% (95% CI, -8.15%, 3.14%), *p* > 0.05; f grade≥3 AST increase: -1.80% (95% CI, -6.71%, 3.11%), *p* > 0.05; g all-grade ALP increase: -3.30% (95% CI, -7.41%, 0.81%), *p* > 0.05;as demonstrated in **Figures 2a–j**. Although we conducted a funnel plot and contour-enhanced funnel plot analysis and performed Egger’s test to assess publication bias, the small number of included studies limited our ability to draw definitive conclusions. Although Egger’s test did not reveal any statistical significance, publication bias could not be ruled out (**Supplementary Figure 1**, **Supplementary Table 1**).

**Figure 3:**
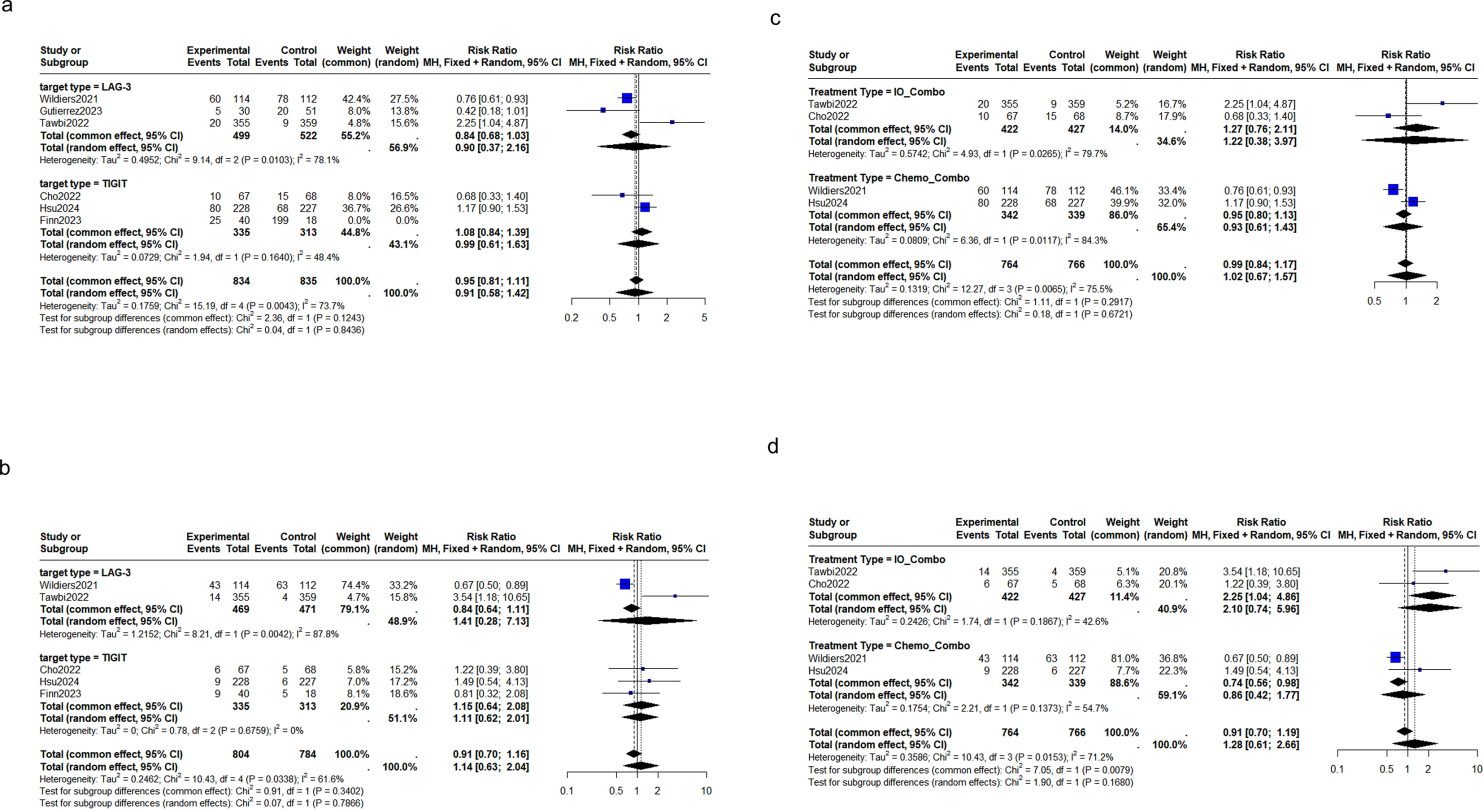
LAG-3 vs TIGIT Inhibitors: Subgroup Analysis of hepatic adverse events in RCTs; **(a)** all-grade hepatic adverse events, **(b)** grade ≥3 hepatic adverse events; Immunotherapy Combination vs chemotherapy Combination: Subgroup Analysis of hepatic adverse events in RCTs: **(c)** all-grade hepatic adverse events, **(d)** grade ≥3 hepatic adverse events(IO_*Combo: Immunotherapy Combination, Chemo*_Combo: chemotherapy Combination).

Subgroup analyses were performed to address significant heterogeneity observed in the initial results. Target-specific analysis revealed no statistically significant differences in hepatic adverse events between LAG-3 and TIGIT inhibitors for all- or high-grade events (**Figure 3**). The treatment modality subgroup analysis results showed some differences between immunotherapy combinations and chemotherapy combinations in their effects on all-grade hepatic adverse events (1.22 [95% CI: 0.38-3.97] *vs*. 0.93 [95% CI: 0.61-1.43]) and high-grade hepatic adverse events (2.10 [95% CI: 0.74-5.96] *vs*. 0.86 [95% CI: 0.42-1.77]). Immunotherapy combinations showed a 22% increased risk of all-grade hepatic adverse events compared to controls (1.22 [95% CI: 0.38-3.97], *p* < 0.05). Chemotherapy combinations showed no significant decrease in the risk of all-grade hepatic adverse events (RR: 0.93 [95% CI: 0.61-1.43], *p* < 0.05). Neither immunotherapy nor chemotherapy combinations showed statistically significant effects on high-grade hepatic adverse events (**Figure 3**).

### The incidence of all-grade and high-grade (≥3) ALT and AST elevation

**Figure 4:**
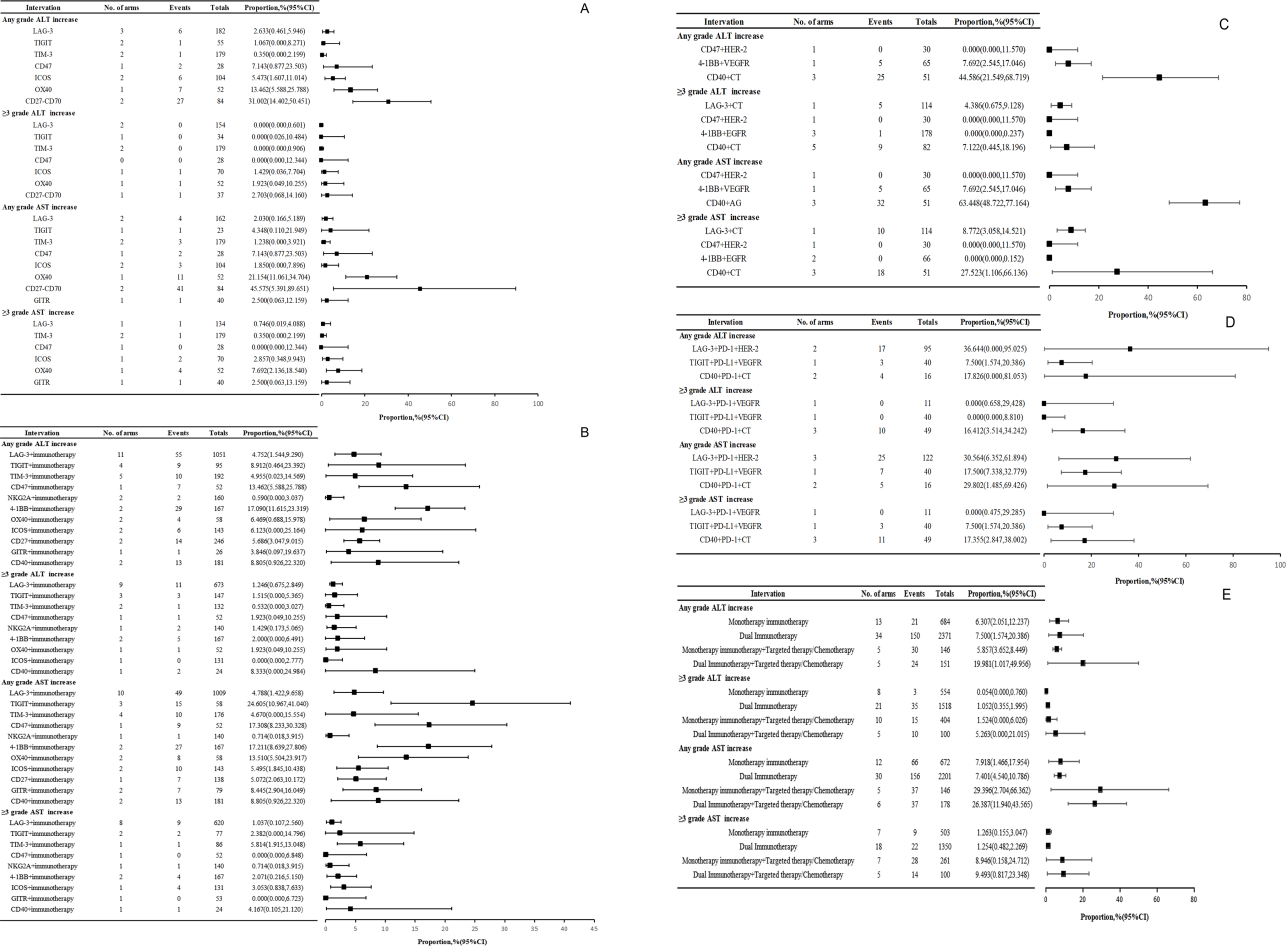
Prevalence of elevated ALT and AST in clinical studies: **(A)** Incidence of all-grade and high-grade ALT and AST increases in monotherapy immunotherapy; **(B)** Incidence of all-grade and high-grade ALT and AST increases in dual immunotherapy; **(C)** Incidence of all-grade and high-grade ALT and AST increases in monotherapy immunotherapy combined with targeted treatments or chemotherapy; **(D)** Incidence of all-grade and high-grade ALT and AST increases in dual immunotherapy combined with targeted treatments or chemotherapy; **(E)** Incidence of all-grade and high-grade increases in ALT and AST in various treatment modalities.

This study included 12 randomized controlled trials and 51 single-arm studies. In the RCTs, ALT/AST elevation occurred in 11 treatment arms involving 574 participants. Single-arm studies reported ALT/AST elevation in 49 treatment arms comprising 2,955 participants. The incidence of all-grade and grade ≥3 ALT/AST elevation was analyzed according to both drug class and treatment modality, as detailed in **Supplementary Figures 2**–**4**. Owing to the paucity of studies, liver-related adverse events were assessed in aggregate from both single-arm and randomized controlled trials, as shown in **Figure 4**.

In monotherapy, CD27-CD70-targeted agents were associated with elevated incidence rates of all-grade and high-grade ALT elevations. OX40-targeted therapy increased the frequency of high-grade AST elevation (**Figure 4A**). Among the combination therapy, 41bb-targeted therapies had higher rates of all-grade liver enzyme elevation, and CD40-targeted combinations exhibited an elevated incidence of high-grade liver enzyme elevation.

Combination therapies with CD40 agonists and chemotherapy resulted in all-grade transaminase elevation rates of 44.49%–63.45% and high-grade rates of 7.12%–27.52%. In contrast, combinations of CD47 and 4-1BB agonists with targeted therapies showed all-grade rates of 0–7.69% and no high-grade events (**Figure 4B**). In dual-immune combinations with targeted therapies or chemotherapy, all-grade transaminase elevation rates ranged from 7.50% to 36.64%, while high-grade rates varied from 0% to 17.83%.

For monotherapy immunotherapy, all-grade transaminase elevation rates ranged from 6.31% to 7.93%, with high-grade elevations ranging from 0.05% to 1.26%. Dual immunotherapy shows all-grade rates of 7.40%–7.50% and high-grade rates of 1.05%–1.25%. When monotherapy is combined with another immunotherapy or chemotherapy, all-grade elevations range from 5.86% to 29.40%, and high-grade elevations from 1.52% to 8.95%. Dual immunotherapy with additional immunotherapy or chemotherapy results in 19.98%–26.38% for all-grade and 5.26%–9.49% for high-grade tumors (**Figures 4C–E**).

### The incidence of all-grade and high-grade(≥3) grade ALP and GGT elevation

**Figure 5:**
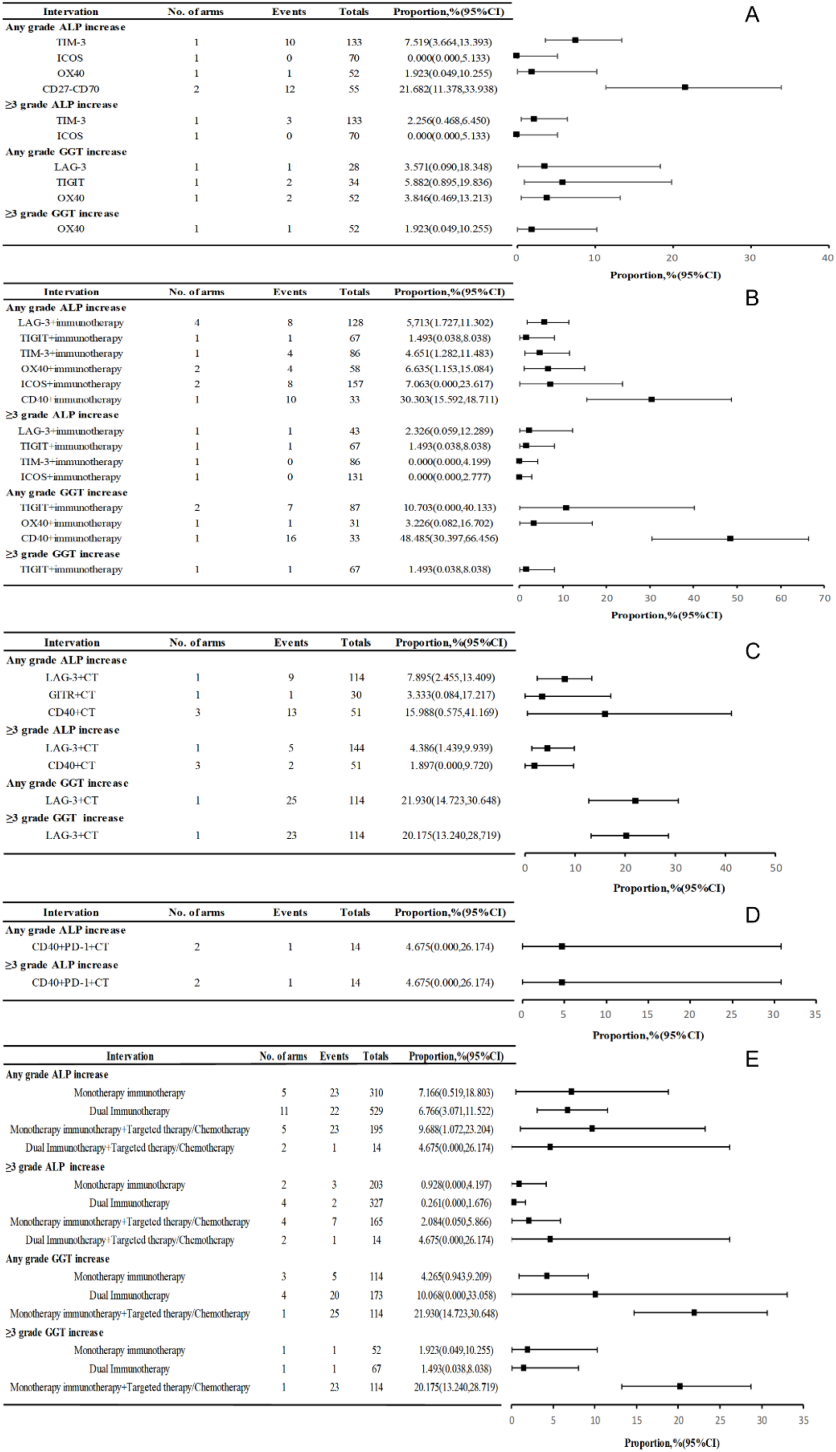
Prevalence of elevated ALP and GGT in clinical studies: **(A)** Incidence of all-grade and high-grade ALP and GGT increases in monotherapy immunotherapy; **(B)** Incidence of all-grade and high-grade ALP and GGT increases in dual immunotherapy; **(C)** Incidence of all-grade and high-grade ALP and GGT increases in monotherapy immunotherapy combined with targeted treatments or chemotherapy; **(D)** Incidence of all-grade and high-grade ALP and GGT increases in dual immunotherapy combined with targeted treatments or chemotherapy; **(E)** Incidence of all-grade and high-grade increases in ALP and GGT in various treatment modalities.

Among the 23 treatment cohorts, 69 patients experienced all-grade ALP elevation. Monotherapy with TIM-3, ICOS, OX40, or CD27-CD70 modulators resulted in a 7.17% incidence, peaking at 21.7% for CD27-CD70. Novel agents combined with PD-1/PD-L1/CTLA-4 inhibitors had a 6.8% rate, while single novel agents with targeted therapies or chemotherapy had a rate of 9.7%. Combinations of novel agents, PD-1/PD-L1/CTLA-4 inhibitors, and targeted therapies or chemotherapy showed a 4.7% rate. Four patients had grade ≥3 elevation, with a high incidence of CD40 agonists combined with PD-1 inhibitors and chemotherapy (**Figure 5**). In eight treatment cohorts (401 patients), 100 patients experienced all-grade GGT elevation. The incidence rates were 4.3% for monotherapy, 10.1% for dual immunotherapy, and 21.9% for monotherapy combined with targeted therapy or immunotherapy. Among the monotherapies, TIGIT inhibitors had a high all-grade GGT elevation rate (5.9%). Notably, no data exists on GGT elevation rates for dual immunotherapy combined with targeted therapies or chemotherapy (**Figure 5**). The incidence of biliary enzyme elevation in randomized controlled trials (RCTs) and single-arm studies is presented in **Supplementary Figures 5**, **6**, respectively.

## Discussion

This systematic review and meta-analysis evaluated adverse events associated with therapies targeting costimulatory and coinhibitory molecules, excluding PD-1, PD-L1, and CTLA-4 inhibitors, in patients with solid tumors. Unlike previous studies that focused on PD-1/PD-L1-related AEs, this is the first report in this area. Accurate reporting of hepatic events is critical for assessing the clinical applicability and guiding clinicians. This global analysis enriches the drug management guidelines and provides actionable insights for clinical practice.

Immunotherapy enhances antitumor immunity but can induce immune-related adverse events (irAEs) across multiple organ systems, including hepatitis, pneumonitis, rash, and hypothyroidism (16–18). Hepatitis is a frequent immune-related AE of ICI monotherapy, occurring in 8.6% of cases, with CTLA-4 inhibitors posing a higher hepatotoxicity risk than PD-1 inhibitors (19, 20). A meta-analysis revealed severe hepatitis (grade 3 or higher) in 50.59% of PD-1/PD-L1-related cases (21). Whether the addition of LAG-3 and TIGIT inhibitors increases the risks associated with conventional anti-tumor therapies remains an unresolved question.

Our analysis of six RCTs showed no significant increase in hepatotoxicity with LAG-3 or TIGIT inhibitors, as liver enzymes, such as ALT, AST, and ALP remained stable. Relevant LAG-3 inhibitors include eftilagimod alpha, favezelimab, and relatlimab (indicated for breast cancer, NSCLC, and melanoma), whereas the TIGIT inhibitor tiragolumab is relevant for ESCC and hepatocellular carcinoma. Although **Figure 2j** suggests a marginal increase in high-grade hepatitis risk with LAG-3 or TIGIT inhibitors, the *p*-value of 0.05 indicates insufficient statistical evidence to confirm this difference. Subgroup analyses suggested that LAG-3 or TIGIT inhibitors did not appear to significantly increase the risk of hepatotoxicity, although routine monitoring remains advisable. A recent meta-analysis indicates that the safety profile of the combination therapy with fianlimab and cemiplimab is consistent with previously reported data for cemiplimab monotherapy and other anti-PD-1 regimens, which aligns with our own conclusions. However, compared to anti-PD-1 monotherapy, the addition of a LAG-3 inhibitor was associated with a higher incidence of trAEs and adverse events leading to treatment discontinuation, although no significant difference was observed in hepatotoxicity. This increase in overall toxicity may be attributed to the broader spectrum of monitored adverse events. Importantly, despite the higher frequency of adverse events, they were generally manageable and rarely necessitated treatment discontinuation (22).

The NCT02614833 trial observed a modest increase in all-grade hepatic adverse events with novel immunotherapy combinations (absolute difference <3%), which was likely attributable to concurrent chemotherapy exposure in both the treatment arms. Furthermore, this analysis could not account for the limitations inherent in early phase trials, including small sample sizes, incomplete follow-up, and potential under-reporting of adverse events. This finding emphasizes the need for larger randomized controlled trials to establish the clinical significance of these observations, while current data support these therapies as novel and safe options for the treatment of advanced tumors.

This meta-analysis revealed a notably high incidence of aminotransferase and ALP elevation in CD27-CD70-targeted monotherapy, likely owing to the inclusion of antibody-drug conjugates (ADCs). ADCs, such as gemtuzumab ozogamicin (targeting CD33) and inotuzumab ozogamicin (targeting CD22) carry FDA black-box warnings for hepatotoxicity, emphasizing the need for caution with CD27-CD70-targeted ADCs. When ADCs were excluded, novel monotherapy showed all-grade alanine aminotransferase (ALT) elevations ranging from 2.63% to 13.46%, with high-grade elevations ranging from 0% to 1.92%. The all-grade aspartate aminotransferase (AST) levels varied from 1.85% to 21.15%.

Immune-related hepatitis (IMH), a distinct subtype of DILI associated with tumor immunotherapy, exhibits incidence variability depending on tumor type and therapeutic combinations. The recorded rates of all-grade IMH range from 1% to 15%, whereas high-grade IMH is reported in 1% to 10% of cases (23, 24). These findings align with the hepatotoxicity profiles previously reported for PD-1/PD-L1 and CTLA-4 inhibitors (25). Among the novel monotherapies, OX40-targeting antibodies demonstrated high rates of both all-grade and high-grade transaminase elevation, potentially attributed to compromised baseline liver function in patients with hepatocellular carcinoma (HCC). Of the 52 participants analyzed, 19 had liver cancer, and underlying cirrhosis and limited hepatic reserve may increase the risk of severe DILI and associated mortality (26). Preclinical studies further suggest that αOX40 exacerbates liver injury by inducing pyroptosis of natural killer T (NKT) cells, although additional clinical data are needed to fully assess this risk (27). In contrast, LAG-3 and TIM-3 inhibitors exhibited favorable safety profiles, with transaminase elevation rates of < 3% for all grades and < 0.8% for high-grade events. These data suggest greater reliability of these therapies. Additionally, cholestatic DILI, despite being a less recognized form of liver injury in cancer treatment, poses a substantial risk of chronic or delayed recovery. Insufficient documentation, particularly regarding ALP or GGT elevation, suggests that the true incidence of cholestatic DILI associated with novel agents may be underestimated (28, 29). Further investigation is imperative to elucidate the hepatotoxicity profiles of emerging immunotherapies, particularly OX40-targeted agents, given their higher incidence of transaminase elevation. A previous meta-analysis indicated that combination therapy with nivolumab and ipilimumab led to higher rates of both all-grade and grade 3 immune-related adverse events than nivolumab monotherapy (30). Dual immunotherapy with PD-1/PD-L1 or CTLA-4 inhibitors resulted in all-grade ALT elevations ranging from 0.59% to 17.09% and AST elevations from 0.71% to 24.61%. High-grade elevations were observed in 0% to 8.33% for ALT and 0% to 5.82% for AST. These elevated rates suggest greater liver damage owing to immune overactivation. While most ICI-chemotherapy combinations showed comparable safety to pembrolizumab monotherapy, atezolizumab chemotherapy (AST 12.0%, ALT 10.5%) and ipilimumab chemotherapy (AST 9.8%, ALT 10.7%) carried the highest hepatotoxicity risk (18). Our study found that combining CD40 inhibitors with chemotherapy increased ALT, AST, and ALP elevation rates of all-grade to over 15%. Available data suggests that CD40-targeted immunotherapy combined with chemotherapy may significantly increase the risk of hepatic toxicity, necessitating cautious clinical consideration. However, given the limited number of included studies, these findings should be interpreted with prudence, resulting in relatively wide confidence intervals. Further clinical investigations are required to validate these preliminary observations and establish more precise safety profiles for this combination therapy. These data underline the heightened risk of hepatotoxicity, especially when PD-1/PD-L1 or CTLA-4 inhibitors are combined with chemotherapy, particularly in patients with NSCLC (31). CD40-targeted immunotherapy enhances chemotherapy sensitivity by modulating the tumor extracellular matrix (32–34). This combination has demonstrated efficacy in inducing tumor regression during chemotherapy or immune checkpoint blockade (33, 35, 36). However, CD40 pathway activation increases hepatotoxicity by promoting myeloid and lymphoid cell migration, leading to immune-mediated delayed hepatitis and heightened liver susceptibility to chemotherapy toxicity (37, 38), often presenting as elevated ALP and GGT levels. Close monitoring of liver markers in clinical practice may enable early diagnosis and reduce steroid use and mortality owing to adverse reactions.

ILICI constitutes a distinct category of indirect DILI, with differences in pathogenesis, clinical presentation, and management compared to conventional direct or idiosyncratic DILI (39). Its proposed mechanism, involving T-cell-mediated autoimmunity or off-target toxicity, is not fully elucidated (40). The heterogeneity in hepatic adverse event incidence across studies likely stems from variations in patient demographics, drug dosing/scheduling, combination regimens, and concomitant hepatotoxic medications, complicating causality assessment (41). These overlapping confounders contribute significantly to inter-study heterogeneity.

The absence of global, dedicated guidelines for oncology drug-related liver injury poses major management challenges. Combination immunotherapies inherently increase hepatotoxicity risk. In patients with pre-existing liver disease or HCC, attributing liver injury to a specific agent is difficult, raising the risk of severe outcomes including mortality. Furthermore, predicting individual susceptibility remains imprecise. HCC patients may be at particularly high risk; thus, optimizing baseline liver function prior to initiating targeted therapy and/or ICIs is crucial (42). Early detection and management of ILICI are therefore critical for treatment success (39). Clinicians should note that CTCAE grading may not fully correlate with DILI severity standards; assessment should be context-driven. For complex or severe cases, a MDT including oncologists and hepatologists is essential.

In conclusion, these findings suggest that clinicians should recognize that the incidence of hepatic adverse events varies according to different treatment modalities. In patients with cancer, combining LAG-3 inhibitors or TIGIT inhibitors did not significantly increase the risk of liver-related adverse events. Therefore, physicians should carefully assess the safety of the various treatment combinations to ensure optimal patient monitoring and management. Improved liver function monitoring can help identify and address potential hepatic adverse reactions early, thereby enhancing the overall treatment safety and efficacy.

### Limitations

This meta-analysis focused on novel pre-commercial drugs; however, data from large cohorts are scarce. Most studies were Phase I or II, limiting the reporting of adverse events. Incomplete data from ASCO and ESMO further hinders the analysis, as many studies are incomplete. Single-arm oncology studies dominate, leaving few RCTs on PD-1 inhibitors, chemotherapy, or PD-L1 inhibitors in combination with targeted therapy. The lack of similar RCTs restricts a deeper comparison of liver toxicity between novel and traditional therapies. Publication bias and potential errors in the original studies further limit the generalizability of the results.

## Conclusion

This meta-analysis revealed that combining LAG-3 or TIGIT inhibitors with existing therapies may not significantly increase the risk of hepatic enzyme elevation or hepatitis. However, further large-scale studies are warranted to confirm these preliminary observations. It comprehensively reviews the incidence of treatment-related adverse events associated with new immunotherapies, both alone and in combination with PD-1/PD-L1/CTLA-4 inhibitors, targeted therapies, and chemotherapies. These results are instrumental in clinical practice, assisting physicians in the early detection of trAEs and the application of proper management to optimize clinical outcomes.

## Data Availability

The raw data supporting the conclusions of this article will be made available by the authors, without undue reservation.
