# Correction: Global assessment of hepatic safety in novel immunotherapies: a systematic review and meta-analysis

**DOI:** 10.3389/fimmu.2026.1788767

**Published:** 2026-01-22

**Authors:** Minyan Ye, Yinuo Dong, Xiaoyun Li, Yang Zhi, Yuping Lu, Jieting Tang, Wei Zhong, Xiaohong Lei, Yimin Mao, Sha Huang, Yanyan Song

**Affiliations:** 1 Department of Medical Oncology, Clinical Oncology School of Fujian Medical University, Fujian Cancer Hospital, Fuzhou, Fujian, China; 2 Division of Gastroenterology and Hepatology, Shanghai Institute of Digestive Diseases, National Health Commission (NHC) Key Laboratory of Digestive Diseases, Shanghai Research Center of Fatty Liver Disease, Renji Hospital, School of Medicine, Shanghai Jiao Tong University, Shanghai, China; 3 Department of Hepatology, Hepatology Research Institute, The First Affiliated Hospital, Fujian Medical University, Fujian Clinical Research Center for Liver and Intestinal Diseases, Fuzhou, Fujian, China; 4 Department of Biostatistics, Clinical Research Institute, Shanghai Jiaotong University School of Medicine, Shanghai, China

**Keywords:** advanced effect, immunotherapy, LAG-3, meta - analysis, safety, TIGIT

The order of authors in the author list of the published paper was erroneously given as:

“Minyan Ye^1^, Yinuo Dong^2^, Xiaoyun Li^2,3^, Yang Zhi^2^, Yuping Lu^1^, Jieting Tang^2^, Wei Zhong^2^, Xiaohong Lei^2^, Yanyan Song^4*^, Sha Huang^1*^ and Yimin Mao^2*^”

The correct author list reads:

“Minyan Ye^1^, Yinuo Dong^2^, Xiaoyun Li^2,3^, Yang Zhi^2^, Yuping Lu^1^, Jieting Tang^2^, Wei Zhong^2^, Xiaohong Lei^2^, Yimin Mao^2*^, Sha Huang^1*^ and Yanyan Song^4*^”

The original version of this article has been updated.

The funder “the National Key R&D Program of China, (2022YFC3502101) to “Yimin Mao” was erroneously omitted.

The funder “National Natural Science Foundation of China, (NSFC 82270619, NSFC 82470621) to “Yimin Mao” was erroneously omitted.

The funder “Joint Funds for the Innovation of Science and Technology, Fujian Province, (2021Y9218) to “Sha Huang” was erroneously omitted.

The funder “Natural Science Foundation of Fujian Province, (2023J011296) to “Sha Huang” was erroneously omitted.

The funder “the Scientific Research Foundation of Fujian Cancer Hospital, (F2326Y-YZK04-02) to “Sha Huang” was erroneously omitted.

The original version of this article has been updated.

In the published article, there was a mistake in the **Funding** statement. The funding statement was describe as “The author(s) declared that financial support was not received for this work and/or its publication”.

A correction has been made to the section **Funding**, Page 11:

“This work was supported by the National Key R&D Program of China (2022YFC3502101), National Natural Science Foundation of China (NSFC 82270619, NSFC 82470621), Joint Funds for the Innovation of Science and Technology, Fujian Province (2021Y9218), Natural Science Foundation of Fujian Province (2023J011296), and the Scientific Research Foundation of Fujian Cancer Hospital (F2326Y-YZK04-02).”

The original version of this article has been updated.

