# Supplementary material for: Global assessment of hepatic safety in novel immunotherapies: a systematic review and meta-analysis
Source: Front Immunol. 2026 Jan 12;16:1677998. doi: 10.3389/fimmu.2025.1677998 (PMC12873479; doi:10.3389/fimmu.2025.1677998)
Supplement: Supplementary file 1 [file Table1.docx]

Supp. Fig 1. (a) all grade hepatic adverse events; (b) grade ≥3 hepatic adverse events; (c) all-grade ALT increase; (d) grade ≥3 ALT increase; (e) all-grade AST increase; (f) grade ≥3 AST increase; (g) all-grade ALP increase; (h) grade ≥3 ALP increase; (i) all-grade hepatitis increase; (j) grade ≥3 hepatitis increase

1)Funnel plots


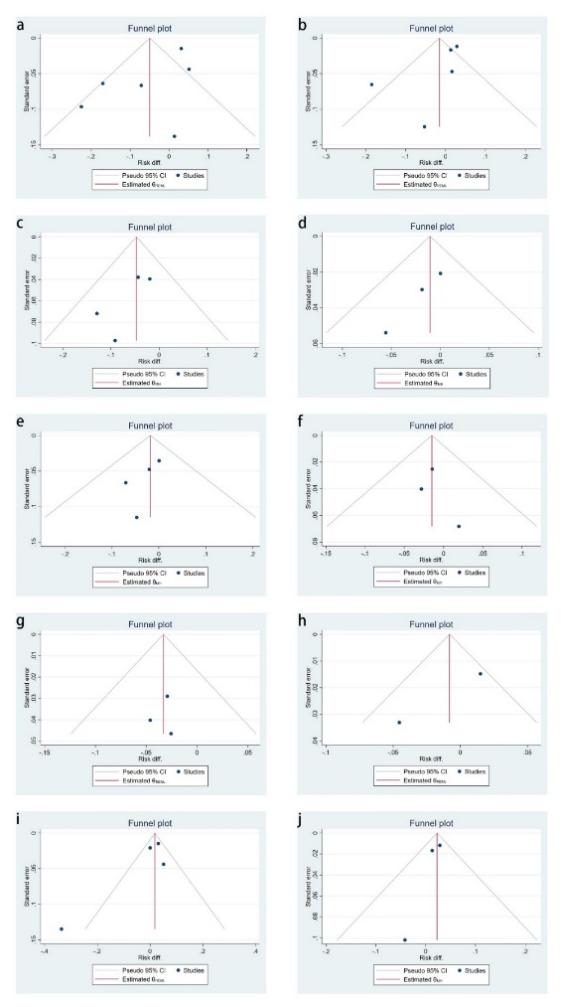


2) Contour-enhanced funnel plots


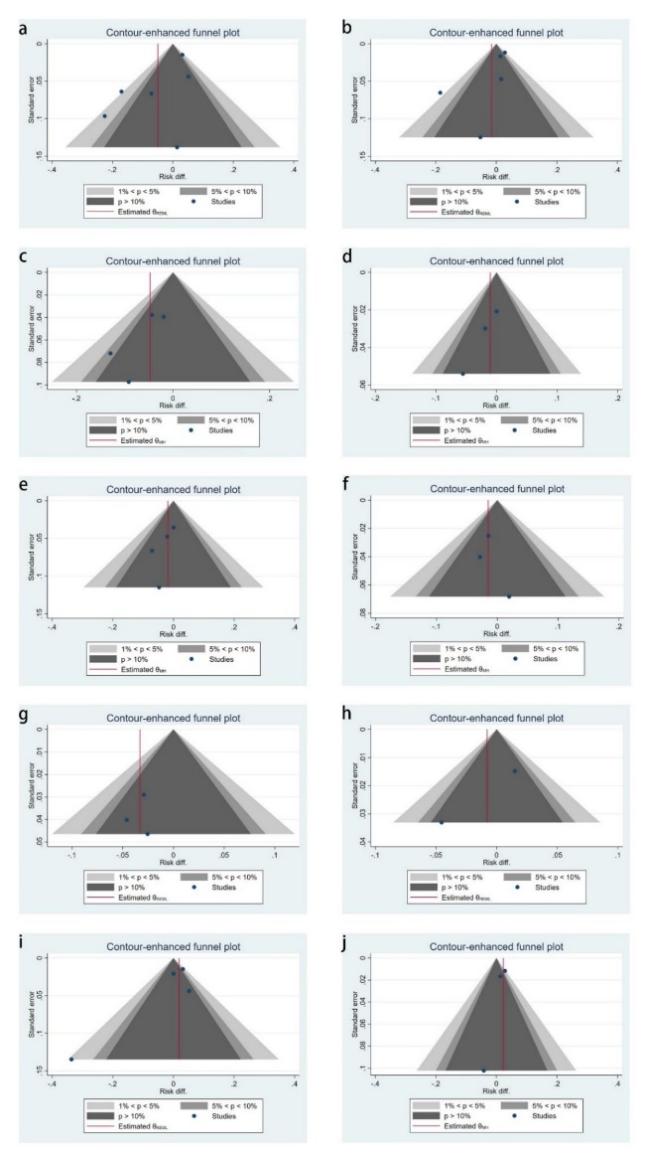


Supp. Fig 2. Prevalence of elevated ALT and AST in RCTs: A Incidence of elevated ALT and AST in response to novel immune-related therapies; B Incidence of elevated ALT and AST in each treatment mode.


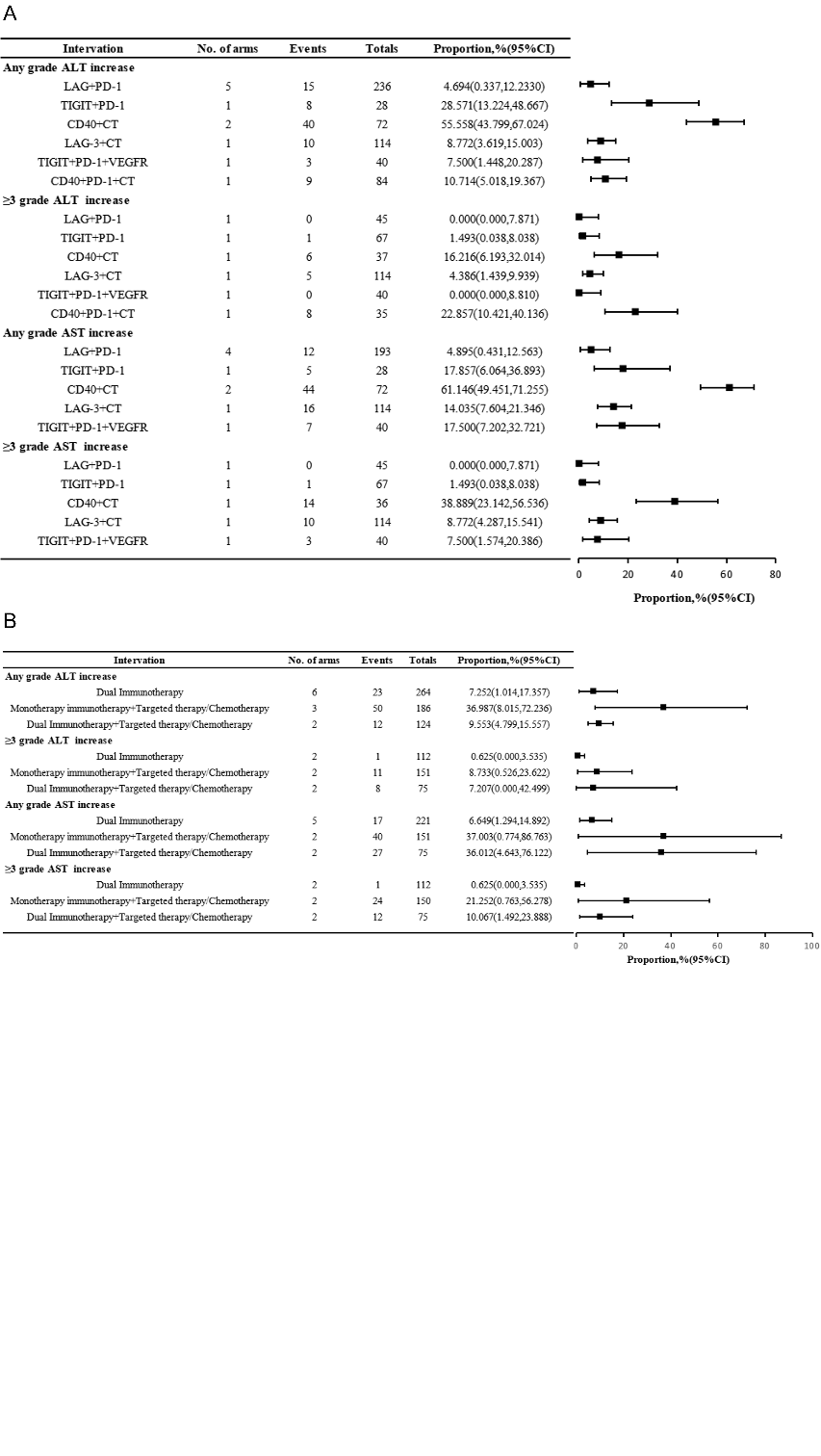


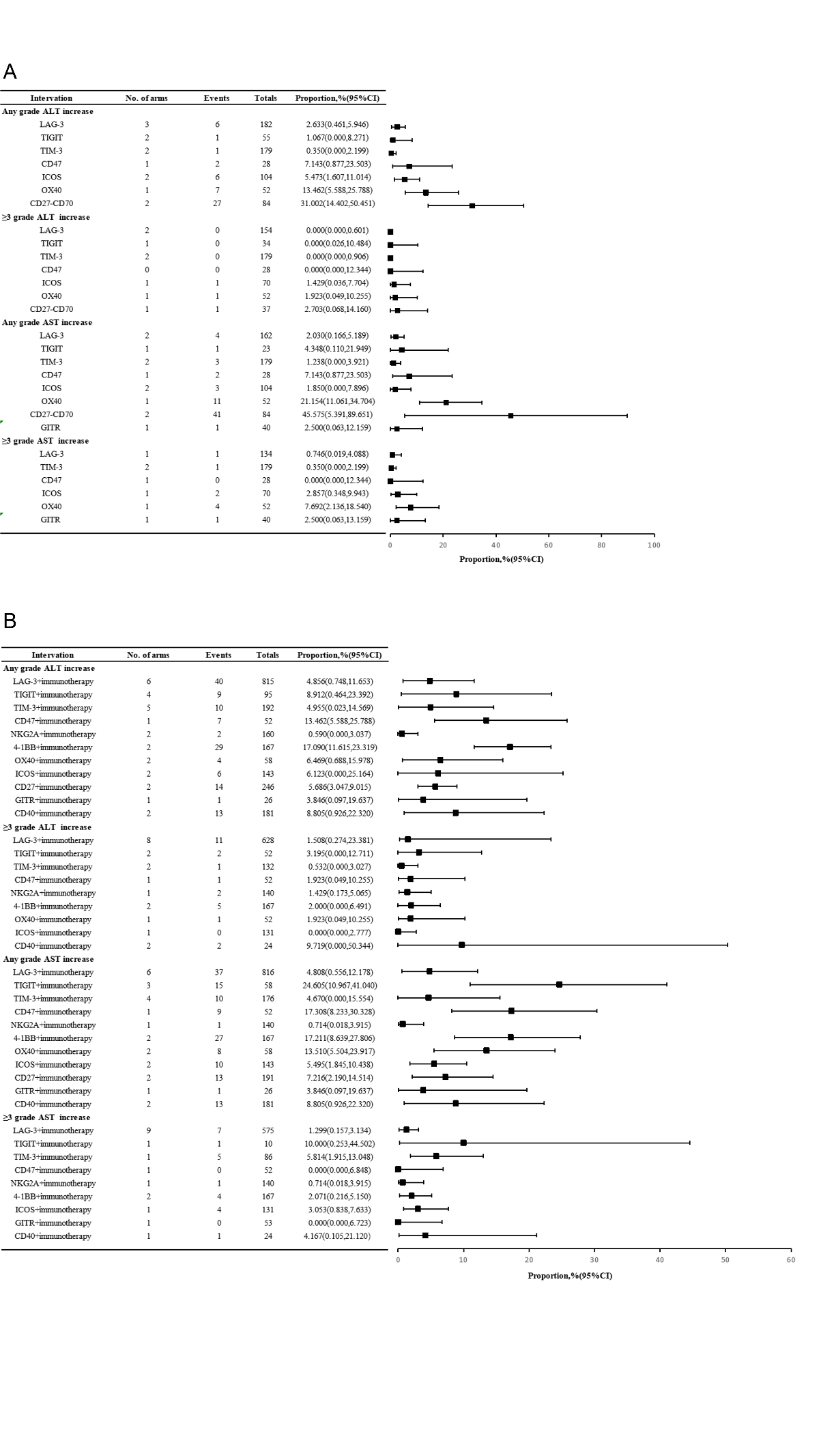
Supp. Fig 3. Prevalence of elevated ALT and AST in single-arm clinical studies: A Incidence of all-grade and high-grade ALT and AST increases in monotherapy immunotherapy; B Incidence of all-grade and high-grade ALT and AST increases in dual immunotherapy.


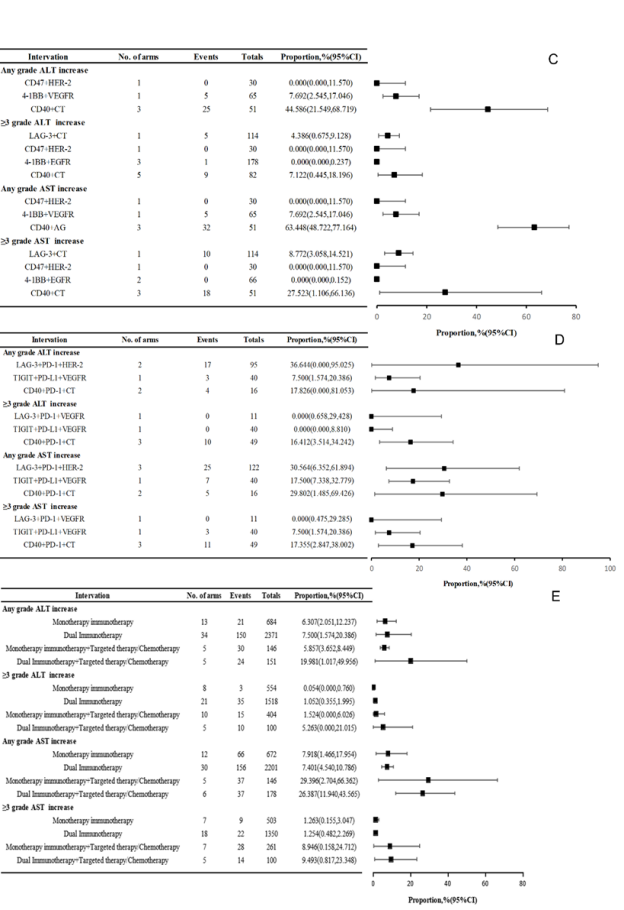
Supp. Fig 4. Prevalence of elevated ALT and AST in single-arm clinical studies: C Incidence of all-grade and high-grade ALT and AST increases in monotherapy immunotherapy combined with targeted treatments or chemotherapy; D Incidence of all-grade and high-grade ALT and AST increases in dual immunotherapy combined with targeted treatments or chemotherapy; E Incidence of all-grade and high-grade increases in ALT and AST in various treatment modalities.

Supp. Fig 5. Prevalence of elevated ALP and GGT in RCTs: A Incidence of elevated ALP and GGT in response to novel immune-related therapies; B Incidence of elevated ALP and GGT in each treatment mode.


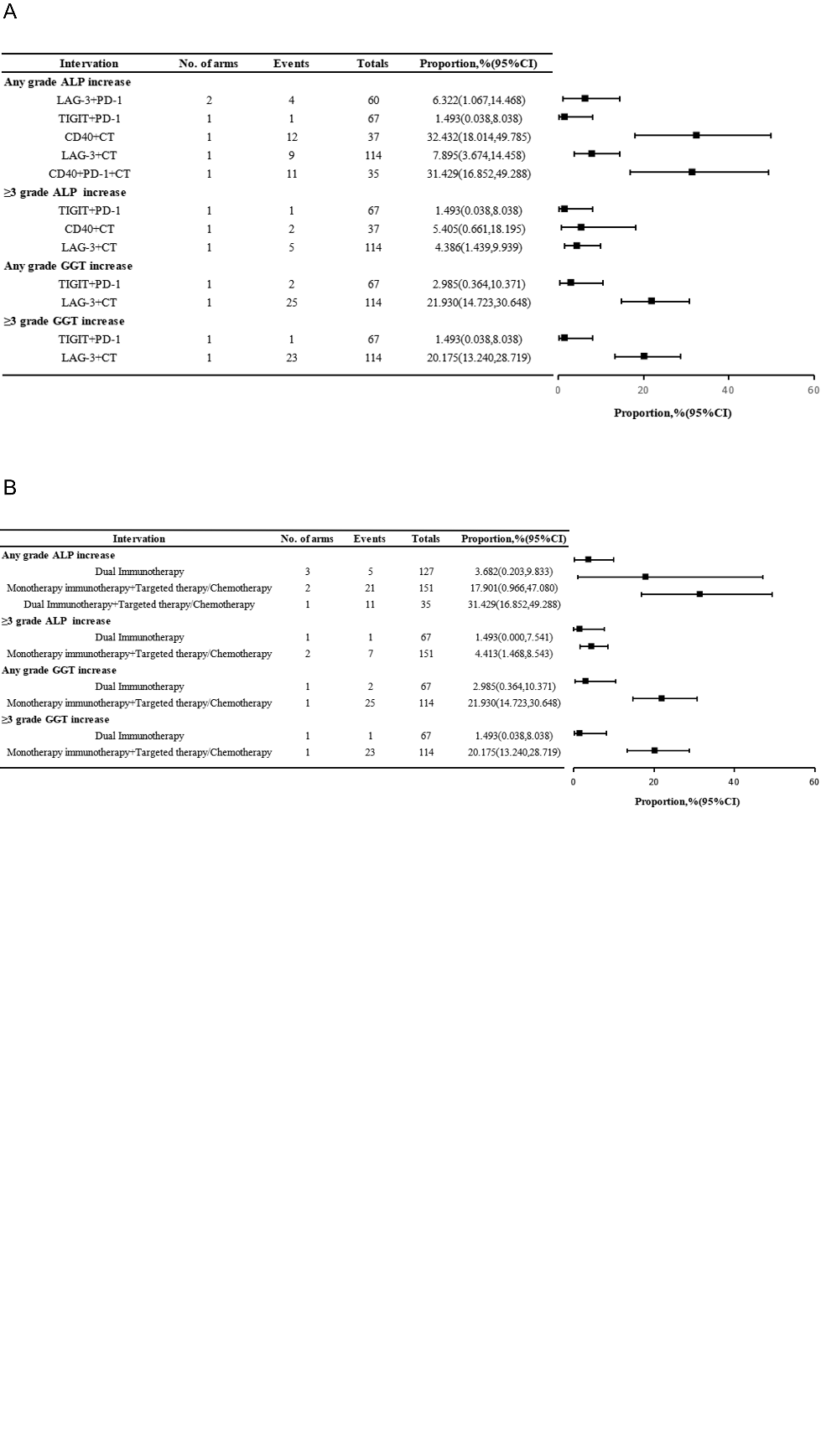


Supp. Fig 6. Prevalence of elevated ALP and GGT in single-arm clinical studies: A Incidence of all-grade and high-grade ALP and GGT increases in monotherapy immunotherapy; B Incidence of all-grade and high-grade ALP and GGT increases in dual immunotherapy; C Incidence of all-grade and high-grade ALP and GGT increases in monotherapy immunotherapy combined with targeted treatments or chemotherapy; D Incidence of all-grade and high-grade ALP and GGT increases in dual immunotherapy combined with targeted treatments or chemotherapy; E Incidence of all-grade and high-grade increases in ALP and GGT in various treatment modalities.


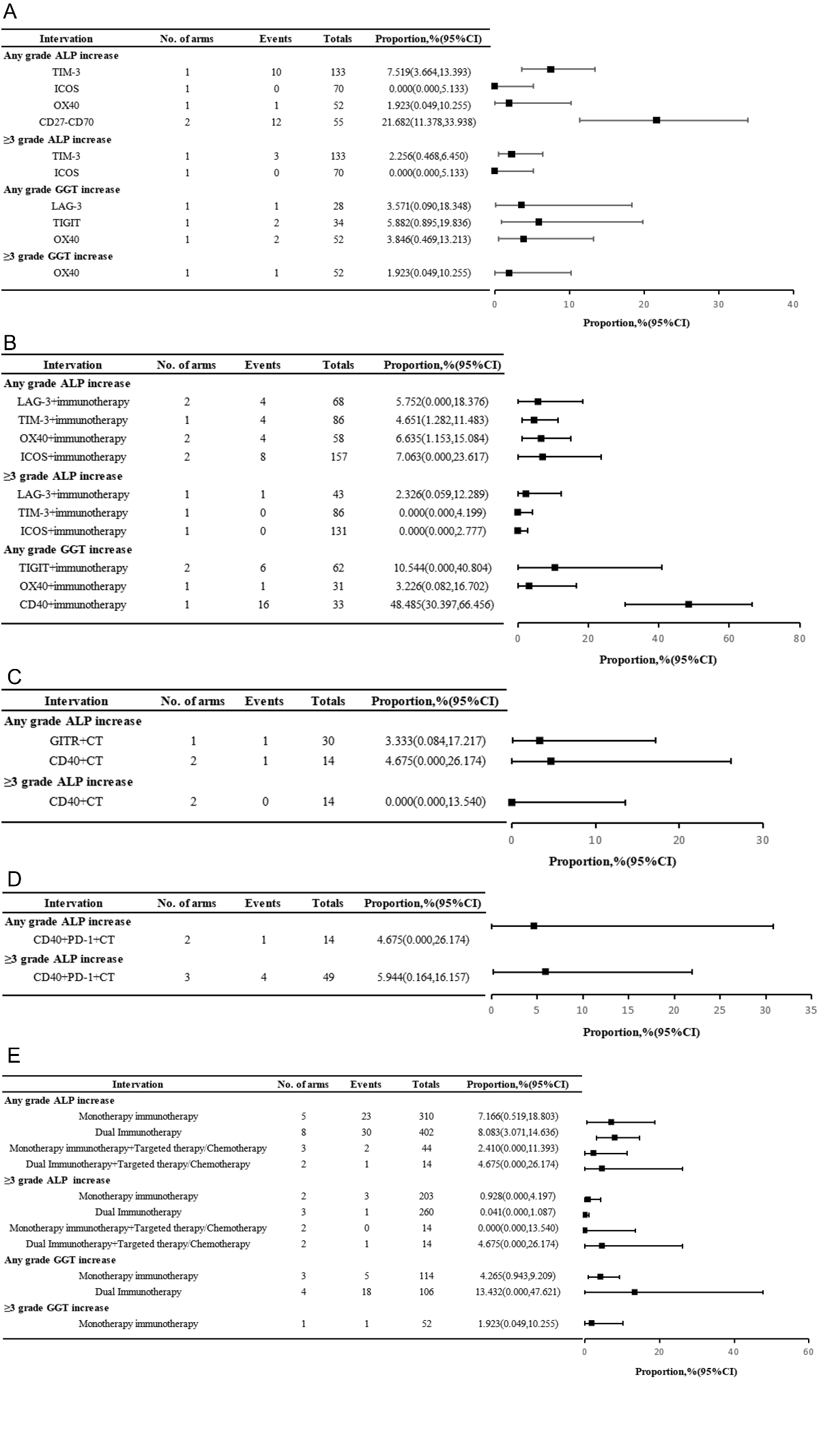


Supp.Table 1. **Egger's test result**

|  | **Kendall's score** | **SE of score** | ***p*** |
| --- | --- | --- | --- |
| **(a) all grade hepatic adverse events** | -5.00 | 5.323 | 0.4524 |
| **(b) grade ≥3 hepatic adverse events** | -6.00 | 4.082 | 0.2207 |
| **(c) all-grade ALT increase** | -2.00 | 2.944 | 0.7341 |
| **(d) grade ≥3 ALT increase** | -3.00 | -3.00 | 0.2963 |
| **(e) all-grade AST increase** | -4.00 | 2.944 | 0.3082 |
| **(f) grade ≥3 AST increase** | 1.00 | 1.915 | 1.0000 |
| **(g) all-grade ALP increase** | -1.00 | 1.915 | 1.0000 |
| **(h) grade ≥3 ALP increase** | / | / | / |
| **(i) all-grade hepatitis increase** | -4.00 | 2.944 | 2.944 |
| **(j) grade ≥3 hepatitis increase** | -1.00 | 1.915 | 1.0000 |

Supp.Table 2.  **Search algorithm and results**

| **PubMed** | **Searching Strategy** | **Results** |
| --- | --- | --- |
| #1 | (clinical trials) AND ((((((((((((((((((((Neoplasms) OR (Tumor)) OR (Neoplasm)) OR (Tumors)) OR (Neoplasia)) OR (Neoplasias)) OR (Cancer)) OR (Cancers)) OR (Malignant Neoplasm)) OR (Malignancy)) OR (Malignancies)) OR (Malignant Neoplasms)) OR (Neoplasm, Malignant)) OR (Neoplasms, Malignant)) OR (Benign Neoplasms)) OR (Benign Neoplasm)) OR (Neoplasms, Benign)) OR (Neoplasm, Benign)))) AND (clinical trials)) | 359479 |
| #2 | ((((((((((((((((((((((((((((((((LAG-3) OR (TIGIT)) OR (TIM-3)) OR (PVRIG)) OR (HHLA2)) OR (VISTA)) OR (BTNL2)) OR (BTN3A1)) OR (BTN2A1)) OR (BTLA)) OR (CD272)) OR (KRLG-1)) OR (CD200R)) OR (NKG2A)) OR (CD47)) OR (B7-H3)) OR (CD276)) OR (B7-H4)) OR (B7S1)) OR (CD160)) OR (TNFRSF9)) OR (4-1BB)) OR (CD137)) OR (OX40)) OR (TNFRSF4)) OR (CD134)) OR (ICOS)) OR (CD278)) OR (CD40)) OR (CD28)) OR (CD27)) OR (GITR)) | 62326 |
| #3 | #1 and #2 | 2106 |
| **Cochrane** | **Searching Strategy** | **Results** |
| #1 | clinical trials' AND 'cancer' OR 'cancers' OR 'malignant neoplasia' OR 'malignant neoplastic disease' OR 'malignant tumor' OR 'malignant tumour' OR 'neoplasia, malignant' OR 'neoplasmic malignancy' OR 'neoplastic malignancy' OR 'oncologic malignancy' OR 'oncological malignancy' OR 'tumor, malignant' OR 'tumoral malignancy' OR 'tumorous malignancy' OR 'tumour, malignant' OR 'malignant neoplasm' | 178455 |
| #2 | LAG-3 or TIGIT or TIM-3 OR PVRIG OR CD112R OR VISTA OR BTNL2 OR BTN3A1 OR BTN2A1 OR BTLA OR CD272 OR KRLG-1 OR CD200R OR NKG2A OR CD47 OR B7H3 OR CD276 OR B7H4 OR B7S1 OR CD160 OR TNFRSF9 OR 41BB OR CD137 OR OX40 OR TNFRSF4 OR CD134 OR ICOS OR CD278 OR CD40 OR CD28 OR CD27 OR GITR | 2704 |
| #3 | #1 and #2 | 791 |
| **Embase** | **Searching Strategy** | **Results** |
| #1 | ('clinical trials'/exp OR 'clinical trials' OR (('clinical'/exp OR clinical) AND trials)) AND ('cancer'/exp OR 'cancer') OR 'cancers'/exp OR 'cancers' OR 'malignant neoplasia'/exp OR 'malignant neoplasia' OR 'malignant neoplastic disease'/exp OR 'malignant neoplastic disease' OR 'malignant tumor'/exp OR 'malignant tumor' OR 'malignant tumour'/exp OR 'malignant tumour' OR 'neoplasia, malignant'/exp OR 'neoplasia, malignant' OR 'neoplasmic malignancy'/exp OR 'neoplasmic malignancy' OR 'neoplastic malignancy'/exp OR 'neoplastic malignancy' OR 'oncologic malignancy'/exp OR 'oncologic malignancy' OR 'oncological malignancy'/exp OR 'oncological malignancy' OR 'tumor, malignant'/exp OR 'tumor, malignant' OR 'tumoral malignancy'/exp OR 'tumoral malignancy' OR 'tumorous malignancy'/exp OR 'tumorous malignancy' OR 'tumour, malignant'/exp OR 'tumour, malignant' OR 'malignant neoplasm'/exp OR 'malignant neoplasm' | 87780 |
| #2 | lag 3' OR tigit OR 'tim 3' OR pvrig OR cd112r OR vista OR btnl2 OR btn3a1 OR btn2a1 OR btla OR cd272 OR 'krlg 1' OR cd200r OR nkg2a OR cd47 OR b7h3 OR cd276 OR b7h4 OR b7s1 OR cd160 OR tnfrsf9 OR 41bb OR cd137 OR ox40 OR tnfrsf4 OR cd134 OR icos OR cd278 OR cd40 OR cd28 OR cd27 OR gitr | 135780 |
| #3 | #1 and #2 | 1799 |
| **Web of science** | **Searching Strategy** | **Results** |
| #1 | clinical trials' AND 'cancer' OR 'cancers' OR 'malignant neoplasia' OR 'malignant neoplastic disease' OR 'malignant tumor' OR 'malignant tumour' OR 'neoplasia, malignant' OR 'neoplasmic malignancy' OR 'neoplastic malignancy' OR 'oncologic malignancy' OR 'oncological malignancy' OR 'tumor, malignant' OR 'tumoral malignancy' OR 'tumorous malignancy' OR 'tumour, malignant' OR 'malignant neoplasm' | 468887 |
| #2 | LAG-3 or TIGIT or TIM-3 OR PVRIG OR CD112R OR VISTA OR BTNL2 OR BTN3A1 OR BTN2A1 OR BTLA OR CD272 OR KRLG-1 OR CD200R OR NKG2A OR CD47 OR B7H3 OR CD276 OR B7H4 OR B7S1 OR CD160 OR TNFRSF9 OR 41BB OR CD137 OR OX40 OR TNFRSF4 OR CD134 OR ICOS OR CD278 OR CD40 OR CD28 OR CD27 OR GITR | 162979 |
| #3 | #1 and #2 | 4443 |

Supp.Table 3. **Baseline characteristics of clinical trials included (n=63)**

| **Study** | **Year** | **Journal/Conference** | **Trial name** | **Trials identifier** | **Phase** | **Name of new agents** | **Study design** | **Enrollment** | **Agents in arms** |  |  |  |  | **Treatment protocols** | | | | |
| --- | --- | --- | --- | --- | --- | --- | --- | --- | --- | --- | --- | --- | --- | --- | --- | --- | --- | --- |
| **LAG-3** |  |  |  |  |  |  |  |  |  | **arm 2** | **arm 3** | **arm 4** | **arm 5** | **arm 1** | **arm 2** | **arm 3** | **arm 4** | **arm 5** |
| Goetze | 2021 | 2021ASCO | - | NCT03252938 | I | Eftilagimod alpha/IMP321 | Cohort study | 12 | Avelumab+Eftilagimod alpha VS Avelumab+Eftilagimod alpha | Avelumab+Eftilagimod alpha | - | - | - | LAG-3+PD-L1 | - | - | - | - |
| Yap | 2023 | CLINICAL CANCER RESEARCH | - | NCT03440437 | I | FS118 | Cohort study | 43 | FS118 | - | - | - | - | LAG-3+PD-L1 | - | - | - | - |
| Lin | 2023 | ONCOIMMUNOLOGY | - | NCT2460224 | II | Ieramilimab | Cohort study | 235 | Ieramilimab+Spartalizumab | - | - | - | - | LAG-3+PD-1 | - | - | - | - |
| Wildiers | 2021 | 2021AACR | - | NCT02614833 | IIb | Eftilagimod alpha/IMP321 | RCT | 114 | Eftilagimod alpha+Paclitaxel | - | - | - | - | LAG-3+CT | - | - | - | - |
| Qiu | 2023 | 2023ASCO | - | NCT04178460 | I | Tebotelimab | Cohort study | 27 | Niraparib+Tebotelimab | - | - | - | - | LAG-3+PD-1+PARP | - | - | - | - |
| Gutierrez | 2023 | ture medicine | KEYNOTE-495/KeyImPaCT | NCT03516981 | II | Favezelimab/MK-4280 | RCT | 81 | Favezelimab+Placebo VS Favezelimab+Placebo | Favezelimab+Placebo | - | - | - | LAG-3+PD-1 | LAG-3+PD-1 | - | - | - |
| Isaacs | 2023 | 2023AACR | I-SPY2 | - | II | REGN3767 | RCT | 430 | REGN3767+Paclitaxel+Cemiplimab+AC | - | - | - | - | LAG-3+PD-1+CT | - | - | - | - |
| Tawbi | 2022 | The New England Journal of Medicine homepage | RELATIVITY-047 | NCT03470922 | II-III | Relatlimab | RCT | 714 | Nivolumab+Relatlimab VS Nivolumab | Nivolumab | - | - | - | LAG-3+PD-1 | PD-1 | - | - | - |
| Deng | 2023 | Therapeutic Advances in  Medical Oncology | - | NCT04414150 | I | SHR-1802 | Cohort study | 28 | SHR-1802 | - | - | - | - | LAG-3 | - | - | - | - |
| Ren | 2024 | 2024ASCO | - | NCT04212221 | I/II | Tebotelimab | Cohort study | 69 | Tebotelimab | - | - | - | - | LAG-3+PD-1 | - | - | - | - |
| Luke | 2023 | nature medicine | - | NCT03219268 | I | Tebotelimab | Cohort study | 353 | Tebotelimab VS Tebotelimab+Margetuximab | Tebotelimab+Margetuximab | - | - | - | LAG-3+PD-1 | LAG-3+PD-1+HER-2 | - | - | - |
| Aigner | 2023 | 2023ASCO | - | NCT04205552 | II | Relatlimab | RCT | 30 | Nivolumab+Relatlimab | - | - | - | - | LAG-3+PD-1 | - | - | - | - |
| Robert | 2021 | 2021ESMO | - | NCT03484923 | II | Ieramilimab | RCT | 45 | Ieramilimab+Spartalizumab | - | - | - | - | LAG-3+PD-1 | - | - | - | - |
| Bai | 2023 | 2023ASCO | - | NCT04640545 | I | LBL-007 | Cohort study | 79 | LBL-007+Toripalimab VS LBL-007+Toripalimab | LBL-007+Toripalimab | - | - | - | LAG-3+PD-1 | LAG-3+PD-1+VEGFR | - | - | - |
| Yang | 2024 | 2024AACR | - | NCT05102006 | Ib/II | LBL-007 | Cohort study | 80 | LBL-007+Toripalimab VS LBL-007+Toripalimab | LBL-007+Toripalimab | - | - | - | LAG-3+PD-1 | LAG-3+PD-1 | - | - | - |
| Felip | 2022 | 2022ASCO | TACTI-002 | NCT03625323 | II | Eftilagimod alpha/TACTI-002 | Cohort study | 114 | Eftilagimod alpha+Pembrolizumab | - | - | - | - | LAG-3+PD-1 | - | - | - | - |
| Garralda | 2022 | ESMO Open | - | NCT02720068 | I | Favezelimab/MK-4280 | Cohort study | 100 | Favezelimab VS Favezelimab+Pembrolizumab | Favezelimab+Pembrolizumab | - | - | - | LAG-3 | LAG-3+PD-1 | - | - | - |
| Timmerman | 2022 | 2022ASCO | - | NCT03598608 | I/II | Favezelimab/MK-4280 | Cohort study | 33 | Favezelimab+Pembrolizumab | - | - | - | - | LAG-3+PD-1 | - | - | - | - |
| Santoro | 2023 | blood | - | NCT03598608 | I/II | Favezelimab/MK-4280 | Cohort study | 25 | Favezelimab+Pembrolizumab | - | - | - | - | LAG-3+PD-1 | - | - | - | - |
| Ascierto | 2023 | Journal of clinical oncology | RELATIVITY-020 | NCT01968109 | I/IIa | Relatlimab | Cohort study | 518 | Nivolumab+Relatlimab VS Nivolumab+Relatlimab | Nivolumab+Relatlimab | - | - | - | LAG-3+PD-1 | LAG-3+PD-1 | - | - | - |
| Schöffski | 2022 | Journal for Immunotherapy of Cancer | - | NCT02460224 | I | Ieramilimab/LAG525 | Cohort study | 255 | Ieramilimab+Spartalizumab VS Spartalizumab | Spartalizumab | - | - | - | LAG-3+PD-1 | LAG-3 | - | - | - |
| **TIGIT** |  |  |  |  |  |  |  |  |  |  |  |  |  |  |  |  |  |  |
| Ying | 2022 | 2022ESMO | - | NCT04672369 |  | IBI939 | RCT | 42 | IBI939+Sintilimab VS Sintilimab | Sintilimab | - | - | - | TIGIT+PD-1 | PD-1 | - | - | - |
| Mettu | 2022 | Clinical Cancer Research | - | NCT03119428 | I a/b | Etigilimab | Cohort study | 33 | Etigilimab+Nivolumab VS Etigilimab | Etigilimab | - | - | - | TIGIT+PD-1 | PD-1 | - | - | - |
| Cho | 2022 | The Lancet Oncology | CITYSCAPE | NCT03563716 | II | Tiragolumab | RCT | 135 | Tiragolumab+Atezolizumab VS Atezolizumab+Placebo | Atezolizumab+Placebo | - | - | - | TIGIT+PD-L1 | PD-L1 | - | - | - |
| Niu | 2021 | Annals of Oncology | MK-7684-001 | NCT02964013 | I | Vibostolimab | Cohort study | 182 | Vibostolimab VS Vibostolimab+Pembrolizumab VS Vibostolimab+Pembrolizumab VS Vibostolimab VS Vibostolimab+Pembrolizumab | Vibostolimab+Pembrolizumab | Vibostolimab+Pembrolizumab | Vibostolimab | Vibostolimab+Pembrolizumab | TIGIT | TIGIT+PD-1 | TIGIT+PD-1 | TIGIT | TIGIT+PD-1 |
| Frentzas | 2023 | Journal for ImmunoTherapy of Cancer | AdvanTIG-105 | NCT04047862 | I/Ib | Ociperlimab | Cohort study | 32 | Ociperlimab+Tislelizumab | - | - | - | - | TIGIT+PD-1 | - | - | - | - |
| Kim | 2023 | JAMA Oncology | - | NCT02794571 | Ia/Ib | Tiragolumab | Cohort study | 73 | Tiragolumab+Atezolizumab VS Tiragolumab | Tiragolumab | - | - | - | TIGIT+PD-L1 | TIGIT | - | - | - |
| Shemesh | 2024 | Cancer Chemotherapy and Pharmacology | CTR20210219/YP42514 | - | I | Tiragolumab | Cohort study | 20 | Tiragolumab+Atezolizumab | - | - | - | - | TIGIT+PD-L1 | - | - | - | - |
| Hsu | 2024 | 2024ASCO GI | SKYSCRAPER-08 | NCT04540211 | III | Tiragolumab | RCT | 461 | Tiragolumab+Atezolizumab+Paclitaxel+Cisplatin | - | - | - | - | TIGIT+CT | - | - | - | - |
| Finn | 2023 | 2023ASCO | MORPHEUS-Liver | NCT04524871 | Ib/II | Tiragolumab | RCT | 58 | Tiragolumab+Atezolizumab++Bevacizumab | - | - | - | - | TIGIT+PD-L1+VEGFR | - | - | - | - |
| **CD40** |  |  |  |  |  |  |  |  |  |  |  |  |  |  |  |  |  |  |
| Vonderheide | 2013 | OncoImmunology | - | NCT00607048 | I | CP-870,893 | Cohort study | 32 | CP-870,893+Paclitaxel+Carboplatin VS Paclitaxel+Carboplatin+CP-870,893 | Paclitaxel+Carboplatin+CP-870,893 | - | - | - | CD40+CT | CD40+CT | - | - | - |
| Nowak | 2015 | Annals of Oncology | - | ACTRN12609000294257 | Ib | CP-870,893 | Cohort study | 15 | CP-870,893+Cis-platinum+Pemetrexed | - | - | - | - | CD40+CT | - | - | - | - |
| Bajor | 2018 | OncoImmunology | - |  | I | CP-870,893 | Cohort study | 24 | CP-870,893+Tremelimumab | - | - | - | - | CD40+CTLA-4 | - | - | - | - |
| O’Hara | 2021 | Lancet Onco | PRINCE, PICI0002 | NCT03214250 | Ib | APX005M/Sotigalimab | Cohort study | 24 | APX005M+Gemcitabine+Albumin-bound paclitaxel VS APX005M+Gemcitabine+Albumin-bound paclitaxel VS APX005M+Gemcitabine+Albumin-bound paclitaxel+Nivolumab VS APX005M+Gemcitabine+Albumin-bound paclitaxel+Nivolumab | APX005M+Gemcitabine+Albumin-bound paclitaxel | APX005M+Gemcitabine+Albumin-bound paclitaxel+Nivolumab | APX005M+Gemcitabine+Albumin-bound paclitaxel+Nivolumab | - | CD40+CT | CD40+CT | CD40+PD-1+CT | CD40+PD-1+CT | - |
| Melero | 2022 | 2022ESMO | - | NCT04083599 | I/II | GEN1042 | Cohort study | 50 | GEN1042+Pembrolizumab VS GEN1042+Pembrolizumab+(Nab-paclitaxel+Gemcitabine or cis/Carboplatin + 5-FU) | GEN1042+Pembrolizumab+(Nab-paclitaxel+Gemcitabine or cis/Carboplatin + 5-FU) | - | - | - | CD40+4-1BB+PD-1 | CD40+4-1BB+PD-1+CT | - | - | - |
| Padrón | 2022 | TURE MEDICINE | - | - | II | APX005M/Sotigalimab | RCT | 99 | Nivolumab+Gemcitabine/Nab-paclitaxel VS Sotigalimab+Gemcitabine/Nab-paclitaxel VS Sotigalimab+Gemcitabine/Nab-paclitaxel+Nivolumab | Sotigalimab+Gemcitabine/Nab-paclitaxel | Sotigalimab+Gemcitabine/Nab-paclitaxel+Nivolumab | - | - | PD-1+CT | CD40+CT | PD-1+CD40+CT | - | - |
| Coward | 2022 | 2022ASCO | - | NCT04481009 | I | YH003 | Cohort study | 20 | YH003+Toripalimab | - | - | - | - | CD40+PD-1 | - | - | - | - |
| Weiss | 2024 | CLINICAL CANCER RESEARCH | - | NCT03123783 | II | APX005M/Sotigalimab | Cohort study | 33 | Sotigalimab+Nivolumab | - | - | - | - | CD40+PD-1 | - | - | - | - |
| **OX40** |  |  |  |  |  |  |  |  |  |  |  |  |  |  |  |  |  |  |
| Kim | 2022 | Clin Cancer Research | - | NCT02219724 | I | MOXR0916 | Cohort study | 172 | MOXR0916 |  |  | - | - | OX40 | - | - | - | - |
| Diab | 2022 | Clin Cancer Research | - | NCT02315066 | I | Ivuxolimab/PF-04518600 | Cohort study | 52 | Ivuxolimab |  |  | - | - | OX40 | - | - | - | - |
| Goldman | 2022 | Clin Cancer Research | - | NCT02705482 | I | MEDI0562 | Cohort study | 58 | MEDI0562+Durvalumab VS MEDI0562+Tremelimumab | MEDI0562+Tremelimumab | - | - | - | OX40+PD-L1 | OX40+CTLA-4 | - | - | - |
| **4-1BB** |  |  |  |  |  |  |  |  |  |  |  |  |  |  |  |  |  |  |
| Segal | 2016 | 2016SITC | - | - | Ib | Urelumab |  | 112 | Urelumab+Cetuximab | - | - | - | - | 4-1BB+EGFR | - | - | - | - |
| Oria | 2022 | 2022ESMO | - | NCT04740424 |  | FS222 | Cohort study | 33 | FS222 | - | - | - | - | 4-1BB+PD-L1 | - | - | - | - |
| Khushalani | 2023 | J Immunother Cancer | - | NCT02110082,NCT02253992 | I/II | Urelumab-3 | Cohort study | 200 | Urelumab+Cetuximab VS Urelumab+Cetuximab VS Urelumab+Nivolumab | Urelumab+Cetuximab | Urelumab+Nivolumab | - | - | 4-1BB+EGFR | 4-1BB+EGFR | 4-1BB+PD-1 | - | - |
| **CD47** |  |  |  |  |  |  |  |  |  |  |  |  |  |  |  |  |  |  |
| Lakhani | 2021 | Lancet Oncology | - | NCT03013218 | - | Evorpacept/ALX148 | Cohort study | 110 | Evorpacept VS Evorpacept+Pembrolizumab VS Evorpacept+Trastuzumab | Evorpacept+Pembrolizumab | Evorpacept+Trastuzumab | - | - | CD47 | CD47+PD-1 | CD47+HER-2 | - | - |
| **NKG2A** |  |  |  |  |  |  |  |  |  |  |  |  |  |  |  |  |  |  |
| Patel | 2023 | Journal for ImmunoTherapy of Cancer | - | NCT02671435 | I/II | Monalizumab | Cohort study | 185 | Durvalumab+Monalizumab VS Durvalumab+Monalizumab | Durvalumab+Monalizumab | - | - | - | NKG2A+PD-L1 | NKG2A+PD-L1 | - | - | - |
| Cascone | 2023 | Cancer Discovery | NeoCOAST | NCT03794544 | II | Monalizumab | RCT | 84 | Durvalumab VS Durvalumab+Monalizumab | Durvalumab+Monalizumab | - | - | - | PD-L1 | PD-L1+NKG2A | - | - | - |
| **TIM-3** |  |  |  |  |  |  |  |  |  |  |  |  |  |  |  |  |  | - |
| Curigliano | 2021 | CLINICAL CANCER RESEARCH | - | NCT02608268 | I/Ib | Sabatolimab/MBG453 | Cohort study | 219 | Sabatolimab+Spartalizumab VS Sabatolimab | Sabatolimab | - | - | - | TIM-3+PD-1 | TIM-3 | - | - | - |
| Harding | 2021 | CLINICAL CANCER RESEARCH | - | NCT03099109 | Ia/b | LY3321367 | Cohort study | 186 | LY3321367 VS LY3321367+LY300054 VS LY3321367 VS LY3321367+LY300054 | LY3321367+LY300054 | LY3321367 | LY3321367+LY300054 | - | TIM-3 | TIM-3+PD-L1 | TIM-3 | TIM-3+PD-L1 | - |
| Falchook | 2022 | 2022ASCO | AMBER | NCT02817633 | I | Cobolimab/TSR-022/GSK4069889 | Cohort study | 108 | Cobolimab | - | - | - | - | TIM-3 | - | - | - | - |
| Acoba |  | NCT03680508 | - | NCT03680508 | II | Cobolimab | Cohort study | 16 | Cobolimab+Dostarlimumab | - | - | - | - | TIM-3+PD-1 | - | - | - | - |
| **CD27-CD70** |  |  |  |  |  |  |  |  |  |  |  |  |  |  |  |  |  |  |
| Tannir | 2014 | Invest New Drugs | - | NCT01015911 | I | SGN-75 | Cohort study | 58 | SGN-75 VS SGN-75 | SGN-75 | - | - | - | CD27 | - | - | - | - |
| Sanborn | 2018 | Jour l for ImmunoTherapy of Cancer | - | NCT02335918 | I/II | Varlilumab | Cohort study | 175 | Varlilumab+Nivolumab VS Varlilumab+Nivolumab | Varlilumab+Nivolumab | - | - | - | CD27+PD-1 | CD27+PD-1 | - | - | - |
| Massard | 2018 | Cancer Chemotherapy and Pharmacology | - | NCT01497821 |  | AMG 172 | Cohort study | 37 | AMG 172 | - | - | - | - | CD70 | - | - | - | - |
| Pal | 2019 | Cancer | - | NCT02216890 | I | SGN-CD70A | Cohort study | 18 | SGN-CD70A | - | - | - | - | CD70 | - | - | - | - |
| Meulenaere | 2021 | Clinical and Translatio l Science | - |  | Ib | Cusatuzumab | Cohort study | 11 | Cusatuzumab VS Cusatuzumab+Chemotherapy | Cusatuzumab+Chemotherapy | - | - | - | CD70 | CD70+CT | - | - | - |
| **GITR** |  |  |  |  |  |  |  |  |  |  |  |  |  |  |  |  |  |  |
| Balmanoukian | 2020 | CLINICAL CANCER RESEARCH | - | NCT02583165 | - | MEDI1873 | Cohort study | 40 | MEDI1873 | - | - | - | - | GITR | - | - | - | - |
| Piha-Paul | 2021 | Jour l for ImmunoTherapy of Cancer | - | NCT02740270 | I/Ib | GWN323 | Cohort study | 92 | GWN323 VS GWN323+Spartalizumab | GWN323+Spartalizumab | - | - | - | GITR | GITR+PD-1 | - | - | - |
| Davar | 2022 | CLINICAL CANCER RESEARCH | TRX518-003 | NCT02628574 | IB | TRX518 | Cohort study | 109 | TRX518 VS TRX518+Gemcitabine VS TRX518+Pembrolizumab VS TRX518+Nivolumab | TRX518+Gemcitabine | TRX518+Pembrolizumab | TRX518+Nivolumab | - | GITR | GITR+CT | GITR+PD-1 | GITR+PD-1 | - |
| **ICOS** |  |  |  |  |  |  |  |  |  |  |  |  |  |  |  |  |  |  |
| Yap | 2022 | CLINICAL CANCER RESEARCH | ICONIC | NCT02904226 | I/II | Vopratelimab/JTX-2011 | Cohort study | 201 | Vopratelimab VS Vopratelimab+Nivolumab | Vopratelimab+Nivolumab | - | - | - | ICOS | ICOS+PD-1 | - | - | - |
| Hilton | 2024 | Cancer Immunology, Immunotherapy | INDUCE-2 | NCT03693612 | I/II | Feladilimab | Cohort study | 26 | Feladilimab+Tremelimumab | - | - | - | - | ICOS+CTLA-4 | - | - | - | - |
| **B7-H3** |  |  |  |  |  |  |  |  |  |  |  |  |  |  |  |  |  |  |
| Shenderov | 2023 | Nat Med | - | NCT02923180 | II | Enoblituzumab | Cohort study | 32 | Enoblituzumab | - | - | - | - | B7-H3 | - | - | - | - |
| Aggarwal | 2022 | Journal for ImmunoTherapy of Cancer | - | NCT02475213 | I/II | Enoblituzumab/MGA271 | Cohort study | 133 | Enoblituzumab +Pembrolizumab | - | - | - | - | B7-H3+PD-1 | - | - | - | - |

Supp.Table 4. **Risk of bias and quality assessment of RCTs included using Jadad scale (n=7)**

| **Study** | **Was the study described as randomized and method of randomization was stated? (Yes: +1; No: 0)** | **Was the method to generate the sequence of randomization was described and it was appropriate? (Yes: +1; Not described: 0; Inappropriate: -1)** | **Was the study described as double blinding (participant and outcome assessor)? (Yes: +1; No: 0)** | **Was there adequate description of the method of masking (eg, identical placebo)? (Yes: +1; Not described: 0; Inappropriate: -1)** | **Was there a description of withdrawals and dropouts for each group and If there were no withdrawals, is there a statement indicating no withdrawal? (Yes: +1; Not described: 0; Inappropriate: -1)** | **Total points (0-5)** |
| --- | --- | --- | --- | --- | --- | --- |
| **Wildiers,2021** | 1 | 0 | 0 | 0 | 0 | 1 |
| **Gutierrez,2023** | 1 | 0 | 0 | 0 | 1 | 2 |
| **Tawbi,2022** | 1 | 0 | 0 | 0 | 1 | 1 |
| **Cho,2022** | 1 | 1 | 1 | 1 | 1 | 5 |
| **Hsu,2024** | 1 | 0 | 0 | 0 | 0 | 1 |
| **Finn,2023** | 1 | 0 | 0 | 0 | 0 | 1 |
| **Ying,2022** | 1 | 0 | 0 | 0 | 0 | 1 |

Supp.Table 5. **Risk of bias and quality assessment of cohort studies included using NOS (n=56)**

| **Study** | **Selection 1** | **Selection 2** | **Selection 3** | **Selection 4** | **Comparability A** | **Comparability B** | **Outcomes 1** | **Outcomes 2** | **Outcomes 3** | **Scores** |
| --- | --- | --- | --- | --- | --- | --- | --- | --- | --- | --- |
|  | **Representat iveness of the exposed cohort** | **Selection of the non- exposed cohort** | **Ascertainment of exposure** | **Demonstration that outcome of interest was not present at start of study** | **Comparability of cohorts on the basis of the design or analysis** | **Study controls for any additional factors** | **Assessment of outcome** | **Follow-up long enough for outcome to occur** | **Adequacy of follow-up of cohorts** | **Total** |
| **Goetze,2021** | * | * | * | * | - | * | * | - | * | 7 |
| **Yap,2023** | * | * | * | * | - | * | * | * | * | 8 |
| **Lin,2023** | * | * | * | * | - | * | * | * | * | 8 |
| **Qiu,2023** | * | * | * | * | - | * | * | - | * | 7 |
| **Isaacs,2023** | * | * | * | * | - | * | * | - | * | 7 |
| **Deng,2023** | * | * | * | * | * | * | * | * | * | 9 |
| **Ren,2024** | * | * | * | * | - | * | * | - | * | 7 |
| **Luke,2023** | * | * | * | * | * | * | * | * | * | 9 |
| **Aigner,2023** | * | * | * | * | - | * | * | - | * | 7 |
| **Robert,2021** | * | * | * | * | - | * | * | - | * | 7 |
| **Bai,2023** | * | * | * | * | - | * | * | - | * | 7 |
| **Yang,2024** | * | * | * | * | - | * | * | - | * | 7 |
| **Felip,2022** | * | * | * | * | - | * | * | - | * | 7 |
| **Garralda,2022** | * | * | * | * | * | * | * | * | * | 9 |
| **Timmerman,2022** | * | * | * | * | - | * | * | - | * | 7 |
| **Santoro,2023** | * | * | * | * | - | * | * | - | * | 7 |
| **Ascierto,2023** | * | * | * | * | * | * | * | * | * | 9 |
| **Schöffski,2022** | * | * | * | * | * | * | * | * | * | 9 |
| **Mettu,2022** | * | * | * | * | * | * | * | * | * | 9 |
| **Niu，2021** | * | * | * | * | * | * | * | * | * | 9 |
| **Frentzas，2023** | * | * | * | * | - | * | * | * | * | 8 |
| **Kim,2023** | * | * | * | * | * | * | * | * | * | 9 |
| **Shemesh，2024** | * | * | * | * | * | * | * | * | * | 9 |
| **Vonderheide，2013** | * | * | * | * | * | * | * | * | * | 9 |
| **Nowak,2015** | * | * | * | * | - | * | * | * | * | 8 |
| **Bajor，2018** | * | * | * | * | - | * | * | * | * | 8 |
| **O’Hara,2021** | * | * | * | * | * | * | * | * | * | 9 |
| **Melero，2022** | * | * | * | * | - | * | * | - | * | 7 |
| **Padrón，2022** | * | * | * | * | * | * | * | * | * | 9 |
| **Coward,2022** | * | * | * | * | - | * | * | - | * | 7 |
| **Weiss,2024** | * | * | * | * | * | * | * | * | * | 9 |
| **Kim,2022** | * | * | * | * | * | * | * | * | * | 9 |
| **Diab,2022** | * | * | * | * | * | * | * | * | * | 9 |
| **Goldman,2022** | * | * | * | * | * | * | * | * | * | 9 |
| **Segal,2016** | * | * | * | * | - | * | * | - | * | 7 |
| **Oria,2022** | * | * | * | * | - | * | * | - | * | 7 |
| **Khushalani,2023** | * | * | * | * | * | * | * | * | * | 9 |
| **Lakhani,2021** | * | * | * | * | * | * | * | * | * | 9 |
| **Patel,2023** | * | * | * | * | * | * | * | * | * | 9 |
| **Cascone,2023** | * | * | * | * | * | * | * | * | * | 9 |
| **Curigliano,2021** | * | * | * | * | * | * | * | * | * | 9 |
| **Harding,2021** | * | * | * | * | * | * | * | * | * | 9 |
| **Falchook,2022** | * | * | * | * | - | * | * | - | * | 7 |
| **Acoba,2023** | * | * | * | * | - | * | * | - | * | 7 |
| **Tannir,2014** | * | * | * | * | * | * | * | * | * | 9 |
| **Sanborn,2018** | * | * | * | * | * | * | * | * | * | 9 |
| **Massard,2018** | * | * | * | * | * | * | * | * | * | 9 |
| **Pal,2019** | * | * | * | * | * | * | * | * | * | 9 |
| **Meulenaere,2021** | * | * | * | * | * | * | * | * | * | 9 |
| **Balmanoukian,2020** | * | * | * | * | - | * | * | * | * | 8 |
| **Piha-Paul,2021** | * | * | * | * | * | * | * | * | * | 9 |
| **Davar,2022** | * | * | * | * | * | * | * | * | * | 9 |
| **Yap,2022** | * | * | * | * | - | * | * | * | * | 8 |
| **Hilton,2024** | * | * | * | * | - | * | * | * | * | 8 |
| **Shenderov,2023** | * | * | * | * | * | * | * | * | * | 9 |
| **Aggarwal,2022** | * | * | * | * | * | * | * | * | * | 9 |
